# NK-A 17E-233I: a novel competitive inhibitor of human dihydroorotate dehydrogenase (DHODH) for cancer therapy

**DOI:** 10.1186/s13046-025-03538-w

**Published:** 2025-10-17

**Authors:** Mohammed Moustapha Anwar, Salvador Meseguer, Néstor García-Rodríguez, Ewa Krupinska, Céleste Sele, Aida Rodríguez-Jiménez, Suraj Verma, Samna Sagadevan, Javier Ramon, Ramon Martí, Annalisa Occhipinti, Claudio Angione, Paloma Ordóñez-Morán, Wolfgang Knecht, Pablo Huertas, M. Angeles Juanes

**Affiliations:** 1 Cytoskeletal Dynamics in Cell Migration and Cancer Invasion Lab, Department of Cancer, Centro de Investigación Príncipe Felipe (CIPF), Valencia, 46012 Spain; 2 Department of Genome Biology, Centro Andaluz de Biología Molecular y Medicina Regenerativa (CABIMER), Universidad de Sevilla-CSIC-Universidad Pablo de Olavide-Junta de Andalucía, Seville, Spain; 3 Department of Biology & Lund Protein Production Platform & Protein Production Sweden, Lund University, Sölvegatan 35, Lund, 22362 Sweden; 4 School of Computing, Engineering and Digital Technologies, Teesside University, Middlesborough, UK; 5 Translational Medical Sciences Unit, School of Medicine, Centre for Cancer Sciences, Biodiscovery Institute, University of Nottingham, Nottingham, UK; 6 Research Group On Neuromuscular and Mitochondrial Diseases, Vall d‘Hebron Research Institute, Autonomous University of Barcelona, Barcelona, Spain; 7 Biomedical Network Research Centre On Rare Diseases (CIBERER), Instituto de Salud Carlos III, Barcelona, Spain; 8 National Horizons Centre, Teesside University, Darlington, UK; 9 Science for Life Laboratory, Department of Biology & Lund Protein Production Platform & Protein Production Sweden, Lund University, Sölvegatan 35, Lund, 22362 Sweden

**Keywords:** NK-A 17E-233I, DHODH, Pyrimidine de novo synthesis, Cancer

## Abstract

Human dihydroorotate dehydrogenase (DHODH) is the rate-limiting enzyme in pyrimidine de novo synthesis and represents a promising target for cancer therapy. However, current inhibitors of DHODH have limited clinical effectiveness and adverse effects. Herein, we report NK-A 17E-233I, a novel small-molecule inhibitor of the human DHODH enzyme, identified through a prospective virtual screening methodology. Molecular docking and biochemical assays show NK-A 17E-233I functions as a pure or partial competitive inhibitor with respect to the natural substrate, dihydroorotate (DHO). It adopts a distinct binding mode from classical inhibitors that target the flavin mononucleotide (FMN) binding cavity of the hydrophobic tunnel. NK-A 17E-233I exhibits selective cytotoxicity in both human cancer cell lines and patient-derived intestinal organoids, inducing DNA damage, S-phase arrest, and cell death. Unlike Brequinar, NK-A 17E-233I preserves mitochondrial respiration via complexes I and II and maintains ATP-linked basal respiration, avoiding the impairment of the electron transport chain (ETC). Our findings imply the aptitude of NK-A 17E-233I as a novel competitive inhibitor of human DHODH, representing a significant advancement in this field since the 1990s.

**Graphical Abstract:**

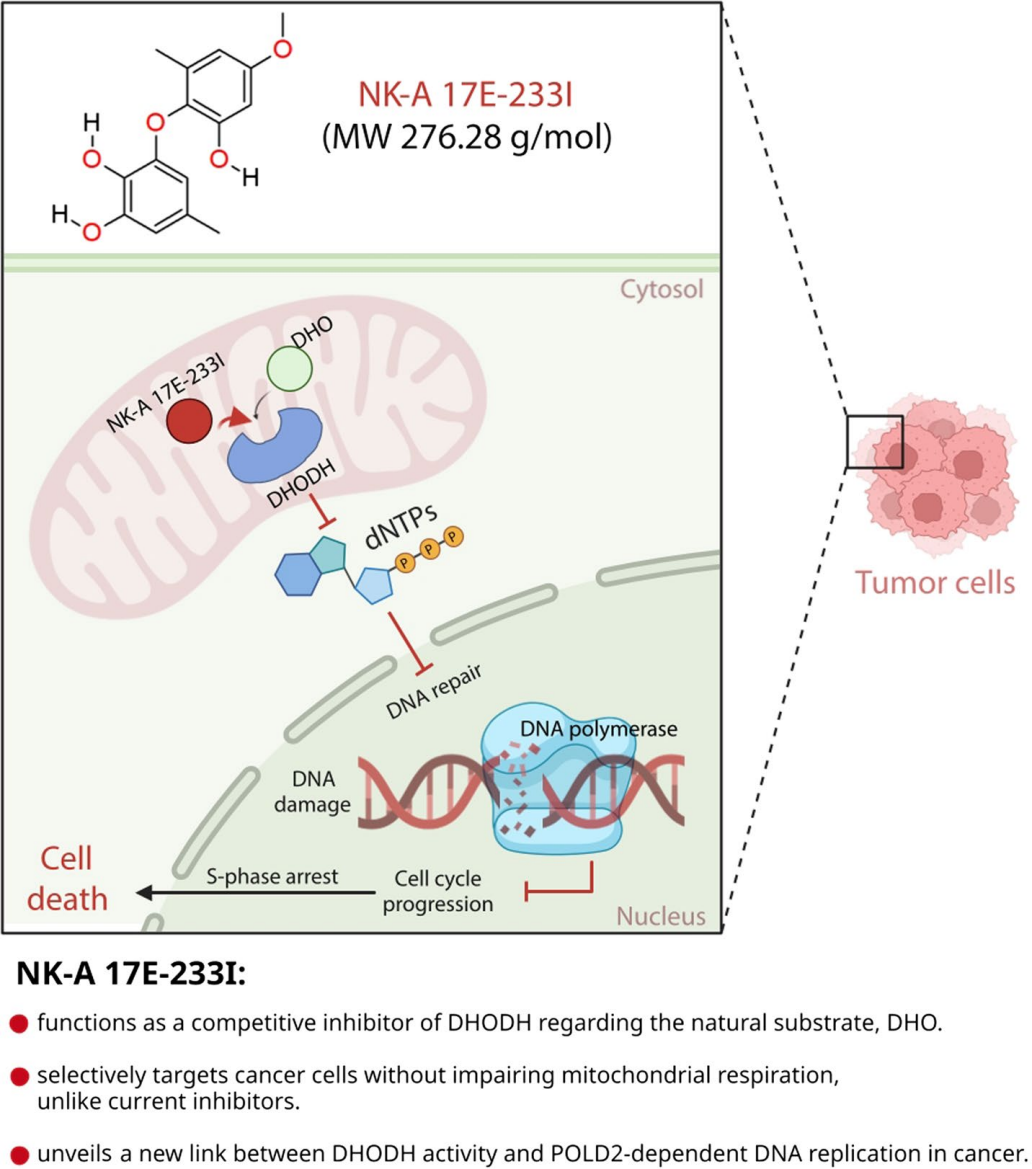

**Supplementary Information:**

The online version contains supplementary material available at 10.1186/s13046-025-03538-w.

## Introduction

The clinical application of the inhibitors of pyrimidine de novo synthesis in cancer has frequently encountered major obstacles, including limited response rates and the emergence of drug resistance [1]. The biosynthesis of purine and pyrimidine nucleotides—essential building blocks of DNA and RNA—occurs through the de novo and salvage routes [2]. Cancer cells rely on a hyperactive pyrimidine de novo pathway to sustain their rampant proliferation, rendering it a compelling target [2]. The inhibition of pyrimidine de novo synthesis by targeting the dihydroorotate dehydrogenase (DHODH) enzyme has proven effective in suppressing cancer cell proliferation [2].

Dihydroorotate dehydrogenase is the second enzyme in the pyrimidine de novo biosynthesis pathway [3]. In humans, it is the only mitochondrial enzyme in this pathway—located on the outside of the inner mitochondrial membrane [4]. It oxidizes dihydroorotate (DHO) to orotate, which serves as a precursor for uridine monophosphate (UMP) nucleotide, from which uridine nucleotide di and triphosphates are then formed by ATP-dependent kinases [3].

Classical inhibitors of DHODH, such as Brequinar and Teriflunomide (the active metabolite of Leflunomide), are reversible inhibitors that bind within flavin mononucleotide (FMN) binding cavity of the hydrophobic tunnel of DHODH. Notwithstanding the therapeutic potential of the inhibition of DHODH, Brequinar and Teriflunomide demonstrated disappointing clinical outcomes [5–7]. For instance, Brequinar demonstrated limited efficacy and a significant incidence of adverse effects, such as myelosuppression, in patients with metastatic colorectal cancer. [8, 9]. Moreover, both Leflunomide and Teriflunomide are currently approved only as immunosuppressants for multiple sclerosis and rheumatoid arthritis, rather than for cancer therapy [10]. This limited success highlights the need to identify new effective inhibitors of human DHODH, possibly with a different mode of action on DHODH as those mentioned above.

Therefore, the overarching goal of the present study was to identify a novel chemotype amenable to further optimization as a viable inhibitor of human DHODH. We utilized a prospective virtual screening approach, integrating ligand-based and structure-based knowledge of the human DHODH enzyme to increase the odds of discovering potential candidates.

## Methods

### Materials and experimental models

All chemicals and reagents were obtained from commercial suppliers, unless otherwise specified. The cancer cell lines utilized in this study (SW620, HCT116, LS174T, HT-29, A549, U2OS, MCF7, MDA-MB-231, and MDA-MB-468) were acquired from the American Type Culture Collection (ATCC) or Merck and were authenticated through short tandem repeat (STR) analysis. Human Foreskin Fibroblasts (HFF; #CCD-1112Sk) were supplied by Dr. Anna Labernadie from Centro de Investigación Príncipe Felipe (CIPF). The cell lines were cultured in high-glucose Dulbecco’s Modified Eagle’s Medium (DMEM) supplemented with sodium pyruvate and L-Glutamine (#41966029; ThermoFisher Scientific), along with 10% fetal bovine serum (FBS; #F9423; Sigma-Aldrich) and 1% penicillin/streptomycin antibiotics (#15140122; ThermoFisher Scientific). Primary Dermal Fibroblasts (Normal Human Adult, HDFa, #PCS-201–012), and Primary Epidermal Keratinocytes (Normal Human Adult, HEKa, #PCS-200–011) were kindly provided by Dr. Ewa Markiewicz from Hexis Lab Limited, Newcastle. HDFa cells were grown in DMEM/F-12 (#11330032; ThermoFisher Scientific) supplemented with 10% FBS, L-Glutamine (#41966029; ThermoFisher Scientific), and 1% penicillin/streptomycin antibiotics. HEKa cells were grown in EpiLife with Calcium (#MEPI500CA; ThermoFisher Scientific) supplemented with Human Keratinocyte Growth Supplement (HKGS; #S0015; ThermoFisher Scientific). Basal forebrain cholinergic neurons derived from mouse embryos were generously provided by Dr. Marçal Vilar from the Institute of Biomedicine of Valencia (IBV), isolated, and cultured in NB/B-27 medium [11]. Human colorectal healthy and tumor samples were collected from the NHS Queens Medical Centre Hospital in Nottingham, UK (ethics license: 17/EM/0126). Tumors were obtained from patients diagnosed with moderately differentiated colon adenocarcinoma exhibiting high immune infiltration. Intestinal epithelial cells were isolated from tissues using 25 μg/mL of Liberase (Roche) at 37 ºC for 1 h, followed by several washes with phosphate-buffered saline (PBS) and subsequent embedding in Matrigel (BD Corning). Three-dimensional organoids were expanded as previously described in [12]. Patient-derived intestinal organoids were mechanically dissociated and cultured in triplicate within 30 μL Matrigel domes in 48-well plates. Following polymerization, organoid culture medium was added and replenished every other day. All cell lines and organoids were maintained at 37 °C, 95% humidity, and 5% CO_2_, and were routinely tested for mycoplasma contamination. All experiments conducted in this study utilized dimethyl sulfoxide (DMSO) as a vehicle control.

### Prospective virtual screening

A prospective virtual screening approach (Fig. S1A) was designed to identify a small molecule with inherent biological activity against the human DHODH enzyme in the context of oncology, which is amenable to subsequent chemical optimization. Ligand-based reverse screening was then integrated, representing one of the target deconvolution techniques employed in contemporary phenotypic profiling, which posits that the ‘query molecule’ exhibits bioactivity against a specific protein [13]. The selected molecule(s) were subjected to molecular docking against the crystal structure of human DHODH and thoroughly validated in the laboratory for their anticancer effects, as well as for their binding affinity and inhibitory activity against DHODH.

### Preparation of ligand database and in silico step-wise filtering

The LOTUS database, recognized as one of the largest repositories of natural products, underwent preliminary filtered according to Lipinski’s Rule-of-Five [14, 15]. Additionally, the topological polar surface area (TPSA) (ranging from 0–140 Å^2^) was incorporated, given its significance as a determinant of oral bioavailability [15]. Ligands deemed eligible for further screening were subjected to the following procedures:

1. ***STOPLIGHT.*** Eligible compounds were analyzed using the STOPLIGHT tool to exclude false-positive (nuisance) hits that may exhibit reactivity, form aggregates, or interfere with assay signals. STOPLIGHT employs domestic quantitative structure interference relationship (QSIR) models to accurately predict the assay liabilities of small molecules across six different assays (Fig. S1A,B) [16].
2. ***SwissADME.*** Validated compounds were uploaded to SwissADME to assess their drug-likeness and lead-likeness properties [17] (Fig. S1A,B).
3. ***SwissTargetPrediction.*** Ligands presumed to target the human DHODH protein were selected for further evaluation (Supplementary Data Table S1).
4. ***ProtoPRED.*** The ProtoPRED platform (https://protopred.protoqsar.com/) was employed to analyze the mutagenicity, melting point, and lipophilicity at pH 7.4 (logD7.4) of the selected molecules. Both mutagenicity and melting point are critical factors for distinguishing anticancer drugs, while logD7.4 is particularly relevant for the inhibitors of human DHODH [15, 18].

### Molecular docking

The crystal structure of human DHODH in complex with Brequinar analog (PDB: 1D3G) was obtained from the RCSB Protein Data Bank (PDB). A blind protein–ligand docking was performed using AutoDock 4.0, employing the Lamarckian Genetic Algorithm. The execution of molecular docking scripts and the preparation of ligand topology were facilitated by Cygwin Terminal facilitated the execution of molecular docking scripts and the preparation of ligand topology. Results were clustered with a root mean square deviation (RMSD) tolerance of 2.0 Å across ten independent docking runs for each ligand. The DHODH-ligand complexes were ranked based on the minimum estimated Gibbs free energy of binding (ΔG) and the number of hydrogen bonds. Visualization of the coordinates of the docked human DHODH within a proximity of < 5 Å and the conventional hydrogen bonds stabilizing the ligand–DHODH interaction, was conducted using Chimera v.1.6 and LigPlot^+^ v.2.2.

### Cell viability assay

Cancer cells (7.5 $\times$ 10^3^) were cultured in 96-well plates and treated with DMSO, NK-A 17E-233I, or Brequinar for 48 h and 96 h, respectively, to evaluate the cytotoxicity and determine the half-maximal inhibitory concentration (IC_50_) using the CellTiter-Glo reagent (#G7571; Promega, Madison, Wisconsin, USA), following the manufacturer’s instructions. The same assay was employed to measure the intracellular levels of ATP at various time intervals (1 h, 4 h, 24 h) after treatment with DMSO or NK-A 17E-233I (25 µM) in both the absence and presence of elevated concentrations of exogenous uridine (200 µM). Luminescence readings were recorded with a Victor Multilabel Plate Reader (PerkinElmer Life Sciences, Massachusetts, USA).

### Organoid culture and drug treatment

To evaluate the cytotoxicity and determine the IC_50_ value, organoids were treated with DMSO or NK-A 17E-233I at the indicated concentrations for 96 h. Cell viability was evaluated employing the CellTiter-Glo 3D Cell Viability Assay (#G9683; Promega, Madison, Wisconsin, USA), according to the manufacturer’s instructions. Luminescence was recorded using a BMG FLUOstar OMEGA-A Plate Reader (BMG LABTECH, Ortenberg, Germany), and the IC_50_ values were calculated using nonlinear regression analysis.

### Cell proliferation assay

Cancer cells (7.5 $\times$ 10^3^) were cultured in 60-mm Petri dishes and treated with DMSO or NK-A 17E-233I (25 µM) to evaluate their proliferation at various time intervals (24 h, 48 h, 96 h, 120 h) using the Trypan Blue exclusion assay [19]. Additionally, SW620 cells (0.8 $\times$ 10^6^) were cultured in 60-mm Petri dishes and treated for 24 h with DMSO, Brequinar (100 µM), or NK-A 17E-233I (25 µM) to evaluate their proliferation in the presence of physiological concentrations of uridine (5 μM and 20 μM) employing the same assay.

### Cell morphology studies

Cancer cells (7.5 $\times$ 10^3^) were cultured in 96-well plates and treated with DMSO or NK-A 17E-233I (25 µM) for 48 h. Brightfield images were captured using a Leica DMi8 microscope with a 10X or 20X objective (HC PL FLUOTAR 10X/0.45 or 20X/0.75) equipped with a Leica DFC9000GT camera, and processed with a Leica Application Suite X (LAS X) Life Science Microscope Software (v3.5.5.19976, RRID:SCR_013673).

### Clonogenic assay

Cancer cells (4 $\times$ 10^3^) were cultured in 6-well plates in triplicate and treated with DMSO or NK-A 17E-233I (25 µM) for two weeks. Colonies were subsequently stained with a solution containing 0.5% crystal violet (#B21932.14; ThermoScientific) and 20% ethanol, followed by multiple washes with water. Plates were allowed to air dry overnight and were subsequently scanned using an Epson Perfection V850 Pro scanner (Kamera Express, Spain). The area of the wells occupied by cells was quantified utilizing the ImageJ plugin ColonyArea.

### Flow cytometry-based cell cycle analysis

Cancer cells (3 $\times$ 10^4^) were cultured in 48-well plates and treated with DMSO or NK-A 17E-233I (25 µM) for 24 h. Following treatment, all wells were washed with PBS (#P4417; Sigma-Aldrich), and the cells were collected in Eppendorf tubes. The cells were harvested using 0.25% trypsin (#25200056; ThermoScientific) and 0.02% EDTA (1:1) (#17892; ThermoFisher Scientific) for 2 min, followed by the addition of DMEM. The tubes were then centrifuged at 500 × g for 5 min. The supernatant was discarded, and the pellets were fixed in 70% cold ethanol and stored at -20 °C for at least 1 h. The pellets were resuspended in 1 mL PBS and centrifuged at 500 × g at 4 °C for 5 min. The supernatant was discarded once again, and the pellets were subsequently reconstituted in a solution containing propidium iodide (PI; #25,535–16-4, Merck) and RNase (#N8080119, Applied Biosystems), followed by storage at 4 °C overnight. Cell cycle analysis was conducted using a CytoFLEX flow cytometer (Beckman Coulter; 405 nm laser). Cells were gated based on size and granularity on a forward scatter (FSC)/side scatter (SSC) plot, with cell debris excluded from the analysis. Mean fluorescence intensity from 1 $\times$ 10^4^ cells was recorded. Data analysis was conducted using FlowJo software v10.8.1 (BD Life Sciences).

### Live-cell imaging

To monitor cell cycle progression, cancer cells (7.5 $\times$ 10^3^) were cultured in 96-well plates for 24 h and stained with 1 µM bisBenzimide H 33342 trihydrochloride (Hoechst 33342) (#B2261; Merck) for 30 min. Following staining, cells were treated with either DMSO or NK-A 17E-233I (25 µM) and subjected to time-lapse imaging for another 24 h, with an acquisition time of 1 h between frames. Cells were maintained at 37 °C with 5% CO_2_ and 95% humidity using Okolab chambers and monitored with a Leica DMi8 microscope equipped with a Leica DFC9000GT camera. Images were captured in brightfield and at 405 nm to visualize the nuclei using a 20X objective (HC PL FLUOTAR 20x/0.75). Images were processed using Leica Application Suite X (LAS X) Life Science Microscope Software (v3.5.5.19976, RRID:SCR_013673).

### Serum starvation of cancer cells

Cancer cells (7.5 $\times$ 10^3^) were cultured in a 96-well plate in DMEM (fortified with 10% FBS) for 24 h. Subsequently, the cells were subjected to serum starvation (FBS-free DMEM) and treated with either DMSO or NK-A 17E-233I (25 µM) for an additional 24 h. Cell proliferation was measured using the CellTiter-Glo reagent as previously described.

### DNA fiber assays

Cancer cells were pulse-labeled with 20 µM iododeoxyuridine (IdU) in the dark at 37 °C for 20 min. Subsequently, the cells were washed twice with PBS and then pulse-labeled with 200 µM chlorodeoxyuridine (CldU) in the presence or absence of NK-A 17E-233I (25 µM) in the dark at 37 °C for 1 h. To assess fork restart in cancer cells following treatment, cells were pulse-labeled with 20 µM IdU for 10 min, after which NK-A 17E-233I (25 µM) was either added or no treatment was administered, followed by an additional 1 h of incubation. The cells were then washed twice with PBS and pulse-labeled with 200 µM CldU for 30 min. Following the labeling procedure, cells were washed twice with PBS, scrapped into PBS containing 0.1% Bovine Serum Albumin (BSA), pelleted, and resuspended in PBS with 0.1% BSA at a final concentration of 1.5 $\times$ 10^3^ cells/µL. A volume of 2.5 µL of the cell suspension was spotted onto a positively charged slide and lysed with 7.5 µL of a spreading buffer (200 mM Tris–HCl, pH 7.5; 50 mM EDTA; 0.5% SDS) at room temperature for 8 min. The slides were then tilted at 45 ° to facilitate DNA spreading. After air-drying, the slides were fixed with ice-cold methanol/acetic acid (3:1) for 5 min, air-dried again, and stored at 4 °C.

The slides were rehydrated with PBS, denatured with 2.5 M HCl for 1 h, washed twice with PBS, and blocked with a blocking buffer (3% BSA; 0.1% Triton X-100 in PBS) for 40 min. Subsequently, the slides were incubated with a primary antibody mixture, including mouse anti-5-Bromo-2'-Deoxyuridine (anti-BrdU) recognizing IdU (#347580; Becton Dickinson, dilution 1:250) and rat anti-BrdU recognizing CldU (#6326; Abcam, dilution 1:250), diluted in a blocking buffer within a dark humid chamber at room temperature for 2.5 h.

For silencing, siNT (non-target, D-001810–10-20 On-TARGET plus Non-targeting pool, Dharmacon) (Horizon Discovery, Waterbeach, UK) and siPOLD2 (Smart pool, ON-TARGETplus Human POLD2 (5425) siRNA oligos directed against human POLD2), were used to transfect cells using RNAiMax Lipofectamine Reagent Mix (Life Technologies) according to the manufacturer’s protocol. 48 h post-transfection, the cells were harvested to evaluate the efficiency of gene silencing, which was estimated to be approximately 90% based on western blot analysis, for further experimental applications.

Afterwards, the slides were washed three times with PBS for 5 min each and incubated with secondary antibodies, anti-mouse Alexa Fluor 594 and anti-rat Alexa Fluor 488 (#A11005 and #A11006, respectively; Invitrogen, dilution 1:250), in a blocking buffer at room temperature in a dark humid chamber for 1 h. After three additional washes with PBS and air-drying, the slides were mounted with ProLong Gold Antifade Reagent (#P36930; Invitrogen) and stored at 4 °C until imaging. Images were acquired using an AF6000 Leica Fluorescence microscope equipped with an HCX PL APO 63x (NA = 1.4) oil objective. ImageJ/Fiji software was utilized to measure the fibers with the segmented line tool.

### The neutral comet assay

The assay was conducted as described in [20]. Cancer cells (2–3 $\times$ 10^5^) were cultured in 6-well plates and treated with DMSO or NK-A 17E-233I (25 µM) for 1 or 24 h. After treatment, the cells were harvested and suspended in Comet Low Melting Agarose (LMA; Trevigen). Once the LMA solidified, the glass slides (CometSlide) were placed in a cell lysis buffer at 4 °C overnight. Electrophoresis was performed using a comet assay electrophoresis system (21 V for 40 min). The slides were subsequently washed and stained with SYBR® Gold dye (Invitrogen) in the dark for 30 min. DNA migration was observed using a fluorescence microscope (AF6000, Leica) equipped with a HCX PL APO 63x (NA = 1.4) oil objective. Comet tail lengths were measured with ImageJ/FIJI, analyzing over 100 cells per each condition.

### DNA damage and cytoskeleton immunostaining

To evaluate DNA damage, cancer cells (1 $\times$ 10^3^) were cultured in 8-well collagen IV Ibidi chamber slides (#80,822; Ibidi) for 24 h, treated with DMSO or NK-A 17E-233I (25 µM) for an additional 24 h, and then fixed. For assessing the integrity of the cytoskeleton, neuronal cells (BFCNs) were cultured on coverslips for 14 days, while non-neuronal cells (cancer cells) were cultured on coverslips for 24 h. Both neuronal and non-neuronal cells were treated with DMSO or NK-A 17E-233I (1 µM) for 24 h, fixed, and permeabilized as described in [21]. Samples were then blocked (non-neuronal cells with 3% BSA in PBS-T and neuronal cells with 10% BSA in PBS-T) and incubated overnight at 4 ºC with the appropriate primary antibodies. Following this, samples were washed with PBS-T and incubated for 1 h with Alexa-488 Phalloidin (#A12379; Invitrogen-ThermoFisher Scientific) to visualize F-actin, along with the corresponding secondary antibodies. The cells were mounted with Ibidi mounting medium containing 4',6-diamidino-2-phenylindole (DAPI, #50011; Ibidi) prior to imaging.

The primary antibodies utilized were: anti–α-tubulin at a 1:1000 dilution (#SC-32293; Santa Cruz), anti–γ-H2AX and anti-Rad51 at 1:500 dilution (#ab22551 and #ab133534, respectively; Cell Signaling), and anti–microtubule-associated protein 2 (MAP2) at a 1:1000 dilution (#OSM00149W; ThermoFisher Scientific). The secondary antibodies employed were donkey anti-mouse Alexa Fluor 568 (#A-10037), goat anti-rabbit Alexa Fluor 568 (#A-11011), and goat anti-guinea pig Alexa Fluor 647 (#A-21450) at a 1:1000 dilution (ThermoFisher Scientific).

### Annexin V-FITC/PI double staining for apoptosis detection

Cancer cells (1 $\times$ 10^4^) were cultured in 96-well plates and treated with DMSO or NK-A 17E-233I (25 µM) for 48 h. Cells were harvested as described in the flow cytometry-based cell cycle analysis section. The supernatant was discarded, and the cells were resuspended in 100 μL of cold Annexin buffer (pH 7.4) composed of 18 mL distilled water, 5.5 mg CaCl_2_, 164 mg NaCl, and 2 mL of 100 mM HEPES. A 1:1 mixture of Annexin V (#130–097-928; Miltenyi Biotec) and propidium iodide (PI, 1 mg/mL) was added to all tubes and incubated in the dark at room temperature for 20 min. Annexin buffer was added, and the samples were analyzed using a CytoFLEX within 1 h. CytExpert and FlowJo software were utilized for data acquisition and analysis.

### Detection of cleaved caspase-3 in apoptotic cells by flow cytometry

Cancer cells (3 $\times$ 10^4^) were cultured in 48-well plates and treated with DMSO or NK-A 17E-233I (25 µM) for 48 h. Following treatment, the cells were harvested, and the supernatant was discarded. Cell pellets were resuspended in 4% methanol-free paraformaldehyde (PFA, #28908; Thermo Fisher Scientific) while vortexing and incubated in the dark at room temperature for 15 min. One milliliter of PBS was added to all tubes, followed by centrifugation at 500 × g for 5 min. The supernatant was discarded, and the pellets were permeabilized using 90% cold methanol with incubation on ice for 10 min. Subsequently, 1 mL of 0.5% BSA (#A3059; Sigma-Aldrich) was added, and the samples were centrifuged at 500 × g for 5 min. The supernatant was discarded, and the pellets were resuspended in 0.5% BSA. The anti–caspase-3 (p17, Cleaved-Asp175) primary antibody (#9661; Cell Signaling, dilution 1:800) was used to incubate samples at room temperature for 1 h. Afterwards, 0.5% BSA was added, and the tubes were centrifuged at 500 × g for 5 min. The supernatant was discarded, and the pellets were resuspended in 0.5% BSA. The samples were then incubated with F(ab')2-Goat anti-Rabbit IgG (H + L) Cross-Adsorbed Secondary Antibody, Alexa Fluor 633 (#A-21072; Invitrogen, dilution 1:1000) in the dark at room temperature for 30 min. Following incubation, 0.5% BSA was added, and the tubes were centrifuged at 500 × g for 5 min. The supernatant was discarded, and the pellets were resuspended in PBS. CytExpert and FlowJo software were employed for data acquisition and analysis.

### RNA sequencing (RNASeq)

Cancer cells (2.2 $\times$ 10^6^) were cultured in 100-mm Petri dishes and treated with either DMSO or NK-A 17E-233I (25 µM) for a duration of 48 h. Dry cell pellets were collected from three independent biological replicates and submitted to Novogene for RNASeq analysis (Cambridge, UK). The raw data are available in detail at the Zenodo repository (10.5281/zenodo.14833856).

### Genome-scale metabolic modeling

To investigate the metabolic alterations, specifically in pyrimidine metabolism, induced by NK-A 17E-233I, we utilized the RNASeq data to construct a computational genome-scale metabolic model (GSMM) based on the highly curated Human-GEM model (Supplementary Data Table S2). The raw data are available at the Zenodo repository (10.5281/zenodo.14833856). The model was manually curated by eliminating dead-end metabolites and reactions, as these do not facilitate any fluxes [22]. We subsequently incorporated sink reactions (i.e., reversible reactions that add or remove intracellular metabolites) for the following metabolites: DHO, orotate, glutamine, pyruvate, citrate, NAD, NADH, aspartate, acetyl CoA, ubiquinol, and ubiquinone. This enabled us to analyze the impact of NK-A 17E-233I on these metabolites.

Utilizing an established approach for condition-specific metabolic modeling [23], we constrained the upper and lower bounds of each metabolic flux in the model using our transcriptomics data to generate sample-specific GSMMs, as outlined in Eq. 1 [24]. These bounds restrict the metabolic flux rate of each reaction in the thermodynamically favored direction within the model, based on the expression levels of the genes encoding the respective reactions:

1 $$\begin{array}{c}lb\left(i\right)=lb\left(i\right)*gamma*\left(reaction\_expression(i)\right)\\ ub\left(i\right)=ub\left(i\right)*gamma*\left(reaction\_expression(i)\right)\end{array},$$

where $i$ represents the index of the reaction (*i* = *1,…,n)*, and $lb$ and $ub$ indicate the lower and upper bounds of the associated reaction flux, respectively. The term $reaction\_expression$ represents the normalized gene expression value mapped to the reactions following gene-protein-reaction rules (GPRs) based on established enzyme biochemistry, while $gamma$ is a hyperparameter used to constrain the model based on gene expression values. Consistent with previously published methodologies [25], a default value of 2 was employed for this hyperparameter.

To evaluate the metabolic alterations across all samples (untreated versus treatment), we estimated the minimum and maximum flux rates of all metabolic reactions using Flux Variability Analysis (FVA) following biomass maximization [22]. Flux Variability Analysis was executed utilizing the Gurobi optimizer (Eq. 2).

2 $$\begin{array}{c}min/\;\mathit{max}\mathit\;v_i,\\{subject\;to\;c^Tv}={\alpha f}_{max},\\\begin{array}{c}Sv=0,\\{\mathit{lb}}_i\leq v_i\leq{ub}_i,for\;i=1,\dots,n,\end{array}\end{array}$$

where *S* is the stoichiometric matrix, $c$ is a vector for choosing the objective function $f$ starting from $v$, $v$ is the reaction flux vector, ${f}_{max}$ is the maximum value of *f* obtained by solving a preliminary LP problem to maximize biomass production. The parameter α represents the fraction of the optimal biomass flux enforced during FVA (for this study, α = 1 for optimal biomass flux). In condition-specific flux variability, each reaction flux $i$ is constrained by lower and upper bounds. The metabolic changes were quantified by calculating the fold change of the flux rates between the treated and untreated control groups.

We then employed robust Metabolic Transformation Analysis (rMTA) [26] to identify genetic biomarkers by analyzing the changes in the metabolic flux rate in the NK-A 17E-233I treatment group when specific genes are knocked out. The resulting metabolic changes were monitored, while the untreated controls remained unchanged. Robust Metabolic Transformation Analysis enhances this analysis by incorporating a worst-case scenario evaluation, assessing the benefits of gene knockout under the least favorable conditions to ensure robustness. Furthermore, a minimization of metabolic adjustment (MOMA) method [27] was applied to reduce the overall changes in metabolic fluxes, thereby providing a more accurate prediction of the impact of NK-A 17E-233I. This comparative analysis enables us to isolate the specific effects of gene modifications, ensuring that the observed changes were solely attributed to NK-A 17E-233I.

### Real-time quantitative polymerase chain reaction (RT-qPCR)

Cancer cells (0.8 $\times$ 10^6^) were cultured in 60-mm Petri dishes and treated for 48 h with DMSO or NK-A 17E-233I (25 µM). The pellets were collected, and the total RNA was isolated using TRI Reagent Solution (#T9424; Sigma-Aldrich). The concentration and purity of RNA samples were assessed using a Nanodrop (Nanodrop ND-1000 spectrophotometer, Thermo Fisher) and stored at -80°C. Complementary DNA (cDNA) was generated using MultiScribe Reverse Transcriptase (#A25776; ThermoFisher Scientific), and the resulting cDNA was diluted and analyzed by qPCR using the StepOnePlus Real-Time PCR System (Applied Biosystems) with Power UP SYBR Green Master Mix (#A25741; ThermoFisher Scientific). β-actin was used as the endogenous control gene, and the fold change was calculated using the 2^−ΔΔCT^ method. Data processing was conducted using StepOnePlus Software for Windows, v2.3. All primer sequences were designed using the Primer 3 Plus software, validated by the Primer-BLAST tool, and submitted to Integrated DNA Technologies (IDT, Madrid, Spain). The primer sequences utilized in this study are listed below:

**Table:** 

Gene	Forward Primer	Reverse Primer
*POLD1*	TCGAGAAAACCAAGCAGCTG	ACGGAGTCAGTGTCACCATAC
*POLD2*	TTGCTTTGCTGACCTTGCTC	CGGACACCAGTAGCACAAAC
*POL ε*	TTTCCTGATGCTGAGACAGACC	GCTTGGGGGTGAACTCAAAATC
*POL μ*	ACTGCGAACCTTAGATGACCTC	TGCTGCAGGGCATCTACATC
*POLι*	ACAGCAGCTGCAAAGTGATG	TGATGTGCAAGACGTCAAGC
*POLθ*	AGCATCGACAAATGGCCATC	TTGAATCTGCCCACGATTGC
*Rev1*	TGGGCAGTACCACCTAAAACC	TGTCCAACTCCTGGTAGATTGG
*PMS2*	ACAATGCACGCATGGAGTTG	ACCTCATTCACGAGTCTGCAG
*MLH1*	TGGCACAGCATCAAACCAAG	AGCATGGCAAGGTCAAAGAG
*MSH2*	AAGGCAAAAAGGGAGAGCAG	ACCGCAGACAGTGATGAAAC
*MSH6*	TTGATGACAGCCCAACAAGG	AAGGCTTCATCTGCACGTTG
*β-actin*	CGAGCGCGGCTACAGCTT	TCCTTAATGTCACGCACGATT
*CoxII*	CGATCCCTCCCTTACCATCA	CCGTAGTCGGTGTACTCGTAGGT
*Sdha*	TCTCCAGTGGCCAACAGTGTT	GCCCTCTTGTTCCCATCAAC

### Western blot

Cancer cells (2.2 $\times$ 10^6^) were cultured in 100-mm Petri dishes and treated with DMSO or NK-A 17E-233I (25 µM) for 48 h. Cells were harvested by scrapping into 2 × Laemmli buffer (4% SDS, 20% glycerol, 125 mM Tris–HCl, pH 6.8) and heated at 100 °C for 5 min. A 4 × Laemmli sample buffer containing β-mercaptoethanol was added to the cell lysates. The proteins were separated using sodium dodecyl sulfate polyacrylamide gel electrophoresis (SDS-PAGE) and subsequently transferred to polyvinylidene fluoride (PVDF) membranes (#88518; Thermo Fisher Scientific) following the manufacturer’s protocols. The membranes were blocked at room temperature for 1 h with 5% dried skimmed milk in Tris-buffered saline with Tween-20 (TBS-T) (20 mM Tris–HCl, pH 7.5, 150 mM NaCl, 0.1% Tween-20), and the blots were incubated with the corresponding antibodies at a dilution of 1:1000–1:3000 in a blocking buffer at 4 °C overnight.

Primary antibodies utilized in this study included: anti-human DHODH (#SC-166348; Santa Cruz), anti–CHK1-pS345 (#2348; Cell Signaling), anti-POLD2 (#29765–1-AP; Proteintech), anti-POLD1 (#15646–1-AP; Proteintech), anti-p53 (#SC-126; Santa Cruz), anti-KRAS, clone 4F3 (#SAB1404011; Sigma-Aldrich), anti-GAPDH (#ab9489; Abcam), anti–α-tubulin (#T9026; Sigma-Aldrich), anti-BAX (#SC-23959; Santa Cruz), anti-cytochrome c (#66264–1-IG; Proteintech), anti-DR5 (#3696; Cell Signaling, dilution 1:500), anti–caspase-8 (#SC-56070; Santa Cruz), and anti–caspase-3 (#9662; Cell Signaling, dilution 1:500). The membranes were incubated at room temperature for 1 h with secondary antibodies (diluted 1:3000–5000), either anti-rabbit (#A0545; Sigma-Aldrich) or anti-mouse IgG-horseradish peroxidase-conjugated (#A9044; Sigma-Aldrich), in TBS-T. The images were analyzed and quantified using ImageStudio software (LI-COR) or ImageQuant 10.2 TL analysis software (Cytiva Life Sciences).

We concurrently conducted the following in vitro activity and biophysical tests to assess the efficacy of NK-A 17E-233I against the human DHODH enzyme and the subsequent effects:

### Recombinant human DHODH

The generation of recombinant enzymes (full-length wild-type, the Miller syndrome variant R135C per UniProt accession number Q02127, or R136C as referenced in PDB: 1D3G, and N-truncated Δ29) and their application in thermal stability and enzymatic activity assays are detailed in [28].

### Thermal stability assay by nano-differential scanning fluorimetry (nanoDSF)

The buffer employed in this study consisted of 10 mM Tris–HCl, 100 mM NaCl, adjusted to a pH of 7.4. n-Dodecyl-β-Maltoside (DDM) was utilized at a concentration of 0.6 mM, which is five times the critical micellar concentration. To ascertain the melting temperature (*T*_m_) of DHODH in the presence of Brequinar and NK-A 17E-233I, Brequinar was applied at 100 μM, while NK-A 17E-233I concentrations ranged from 200 µM to 10 µM. Protein unfolding was evaluated based on the ratio of wavelengths recorded at 350 nm and 330 nm (tryptophan/tyrosine shifts) with laser powers set at either 60% or 20%.

### In vitro* activity assay*

The assays to determine the inhibition constants of inhibitors of the recombinant DHODH enzymes are described in [28, 29]. Brequinar and NK-A 17E-233I were solubilized in DMSO to final concentrations of 210 µM and 20 mM, respectively. To determine the IC_50_ value of Brequinar and NK-A 17E-233I, Brequinar and NK-A 17E-233I were varied from 200 nM to 0.01 nM and from 400 µM to 0.8 µM, respectively, in the activity assay using 1 mM L-DHO and 0.1 mM decylubiquinone (Q_D_) as substrates.

In the activity assay utilizing 0.1 mM DHO and 0.1 mM Q_D_ as substrates, Brequinar and NK-A 17E-233I were varied from 1000 nM to 0.01 nM and from 400 µM to 0.8 µM, respectively.

The IC_50_ values were determined by fitting the equation *V* = *V*_max_/(1 + (*I*/IC_50_)^*h*^) to the assay data using SigmaPlot14 (Systat Software Inc.). In this equation, *V* represents the measured enzyme activity, while *V*_*max*_ indicates the maximum enzyme activity reached. *I* denotes the inhibitor concentration, and *h* describes the slope of the fitted curve.

### Determination of intracellular nucleotide concentration, orotate, and related metabolites in total cell extracts

Cancer cells (2.2 $\times$ 10^6^) were cultured in 100-mm Petri dishes and treated with DMSO or NK-A 17E-233I (25 µM) for 24 h. Nucleotides and related compounds were extracted from cell pellets by treatment with 0.5 M trichloroacetic acid (TCA) followed by neutralization and solvent evaporation as detailed in [30]. Dry extracts were stored at -70°C until further analysis. Protein pellets sedimented after TCA precipitation were re-dissolved in 1 mL of 100 mM NaOH, 5% SDS by incubation at 95ºC for 5 min, followed by complete re-dissolution through several syringe strokes with a 23G needle. The concentration of the re-dissolved protein was quantified using the Pierce™ BCA Protein Assay Kit (Thermo Fisher Scientific), with the amount of the metabolites referenced to protein content.

On the day of metabolite analysis, the dry extracts were re-dissolved in 150 µL of water. Nucleotides, DHO and orotate concentrations were determined using HPLC–MS/MS using Acquity UPLC-Xevo™ TQ Mass Spectrometer (Waters, MA, USA), following the methodology described in [30]. The m/z transitions for DHO and orotate were 156.87 > 113.09 and 154.90 > 111.00, respectively. The identification of all compounds was based on retention time and specific ion transitions. The calibration curves generated with aqueous standards were processed concurrently, and the concentrations were derived from peak area interpolation using TargetLynx software (Waters, MA, USA).

### Detection of mitochondrial membrane potential (ΔΨm) and oxidative stress (ROS)

Cancer cells (1 $\times$ 10^3^) were cultured in 8-well collagen IV Ibidi chamber slides and treated with DMSO or NK-A 17E-233I (25 µM) for 24 h. The media was replaced with fresh media containing 100 nM MitoTracker Red dye to measure ΔΨm (1 mM stock; #M7512; Thermo Fisher Scientific) and 5 µM CellROX Deep Red Reagent to assess oxidative stress levels (2.5 mM stock; #C10422; Thermo Fisher Scientific) for 45 min. The cells were then fixed with 4% methanol-free PFA, washed with PBS, and mounted with Ibidi mounting medium containing 4',6-diamidino-2-phenylindole (DAPI) prior to imaging.

### Estimation of mitochondrial respiration

Oxygen consumption rate (OCR) was measured using a high-resolution respirometer (Oxygraph-2k, Oroboros Instruments, Innsbruck, Austria) [31]. SW620 cells (2.2 $\times$ 10^6^) were cultured in 100-mm Petri dishes and treated with DMSO or NK-A 17E-233I (25 µM) for 24 h. Cells were harvested at 80% confluency using 0.25% trypsin/EDTA and resuspended in complete DMEM at a concentration of 5 $\times$ 10^5^ cells/mL. The cells were subsequently analyzed in two 2 mL Oxygraph chambers. Real-time measurements of OCR were conducted at 37 °C under basal conditions and following the sequential addition of inhibitors targeting various mitochondrial respiratory complexes: oligomycin (2.5 μM) to inhibit complex V (to assess ATP-linked respiration and leak rate), carbonyl cyanide-p-trifluoromethoxyphenylhydrazone (FCCP) with stepwise titration in 0.5 μM increments (to evaluate maximal electron transport system respiratory capacity and reserve capacity), and rotenone (0.5 μM) and antimycin A (2.5 μM) to inhibit complex I and complex III, respectively (to measure non-mitochondrial oxygen consumption). The values of OCR were normalized to mitochondrial DNA copy number, which was quantified by qPCR using the primers referenced above. Data analysis was performed using DatLab7 (Oroboros, Austria) software.

To assess complex I- and complex II-dependent respiration, SW620 cells (2.2 $\times$ 10^6^) were cultured into 100-mm Petri dishes and treated with DMSO, NK-A 17E-233I (25 µM), or Brequinar (100 µM) for 24 h. Following treatment, cells were detached using trypsin–EDTA, washed with PBS, and resuspended in MiR05 media (composed of 110 mM sucrose, 60 mM potassium-lactobionate, 0.5 mM EGTA, 3 mM MgCl_2_·6H_2_O, 20 mM taurine, 10 mM KH_2_PO_4_, 20 mM HEPES adjusted to pH 7.1 with KOH at 37 °C, and 1 g/L BSA) at a concentration of 5 $\times$ 10^5^ cells/mL. The cells were then transferred into 2 mL Oxygraph chambers for real-time measurement of OCR. Initial permeabilization was achieved using 7 μg of digitonin, followed by the sequential addition of substrates and inhibitors: 5 mM ADP, 2 mM malate, 10 μM cytochrome c, 5 mM pyruvate, 10 mM glutamate, 10 mM succinate, 0.5 μM rotenone, and 2.5 μM antimycin A. Mitochondrial membrane integrity was confirmed through the addition of cytochrome c (10 μM), with observed changes in OCR remaining below 10%. Complex I-mediated respiration was evaluated following the addition of complex I substrates (malate/pyruvate/glutamate), while complex II-mediated respiration was assessed in the presence of the complex II substrate (succinate) and the complex I inhibitor (rotenone). Residual oxygen consumption after the addition of antimycin A was subtracted from all the results to yield mitochondria-specific rates. Data analysis was performed using DatLab7 (Oroboros, Austria) software. Oxygen consumption rates were expressed as picomoles (pmol) per second per million cells (Mill) and normalized to the mitochondrial copy number in each sample.

Additionally, we evaluated DHODH-dependent respiration in SW620 cells (1 $\times$ 10^6^) treated with DMSO, NK-A 17E-233I (25 µM), or Brequinar (100 µM) for 24 h. Cells were resuspended in MiR05 medium and transferred into the chambers of the Oxygraph-2k instruments. The OCR in digitonin-permeabilized cells was monitored in the presence of the complex I inhibitor (0.5 μM rotenone), 1 mM DHO, 3 mM ADP, 10 μM cytochrome c, and 30 μM Leflunomide. DHODH-mediated respiration was calculated by subtracting the residual respiration rate after the addition of 30 μM Leflunomide from the respiration rate measured in the presence of 0.5 μM rotenone, 1 mM DHO, 3 mM ADP, and 10 μM cytochrome c.

### Lactate assay

SW620 cells (0.8 $\times$ 10^6^) were cultured in 60-mm Petri dishes and subjected to treatment with DMSO, NK-A 17E-233I (25 µM), or Brequinar (100 µM) for a duration of 24 h to evaluate the glycolytic capacity of cancer cells via lactic acid production. This assessment was conducted utilizing a Lactate Assay Kit-WST (#L256-20; Dojindo Laboratories), in accordance with the manufacturer's protocol. Optical density measurements were recorded at 450 nm using a Victor Multilabel Plate Reader.

### Glutathione assay

SW620 cells (0.8 $\times$ 10^6^) were cultured in 60-mm Petri dishes and treated with DMSO, Brequinar (100 µM), or NK-A 17E-233I (25 µM) for 24 h, to quantify the reduced and oxidized glutathione (GSH/GSSG) using a GSH/GSSG Assay Kit (#HY-K0311; MedChemExpress) in accordance with the manufacturer's instructions. Optical density measurements were recorded at 450 nm using a Victor Multilabel Plate Reader.

### Fixed-cell imaging

Images were captured using a Leica DMi8 microscope with a 10X or 20X objective (HC PL FLUOTAR 10X/0.45 or 20X/0.75) equipped with a Leica DFC9000GT camera, utilizing a 15-ms exposure time to obtain image stacks (11 planes, 0.5 µm steps). In some cases, a Leica SP8 confocal microscope with a 63X oil objective (HC APO 63X/1.40 CS2) was employed, equipped with an EL6000 Fl light source, at 1–30% laser power using photomultiplier tube (PMT) and/or hybrid (HyD) detectors in sequential mode, with a pinhole setting of 1.00 AU. Images were analyzed using Leica Application Suite X (LAS X) Life Science Microscope Software (v3.5.5.19976, RRID:SCR_013673).

### Fluorescence intensity measurements

The density of γ-H2AX, Rad51, ΔΨm, and oxidative stress in cancer cells was measured as described in [32], utilizing custom macros available as open access at Zenodo (https://zenodo.org/records/8387899) to obtain raw intensities and individual cell area. The intensity/area (i.e., density) ratio per cell was subsequently plotted in GraphPad Prism (v10.3.1; GraphPad Software, La Jolla, CA), and displayed in bar charts, violin plots, or as Superplots [33, 34].

### Statistical analysis

Statistical analyses were conducted using GraphPad Prism (v10.0.0 (153), RRID:SCR_002798). Figure legends provide comprehensive details on the number of replicates (n), error metrics, and the statistical tests employed in each experiment. Superplots illustrate the mean 'N' of the various replicates while also displaying the distribution of 'n' as color-coded dots within a violin plot. Data are presented as mean ± standard error of the mean (SEM) or standard deviation (SD), as specified in each figure legend. Only the data in Table 2 are expressed as median [Minimum – Maximum]. Statistical significance: n.s., not significant; * p < 0.05; ** p < 0.01; *** p < 0.001; *** p < 0.0001.

## Results

### NK-A 17E-233I is a novel anticancer compound

**Fig. 1:**
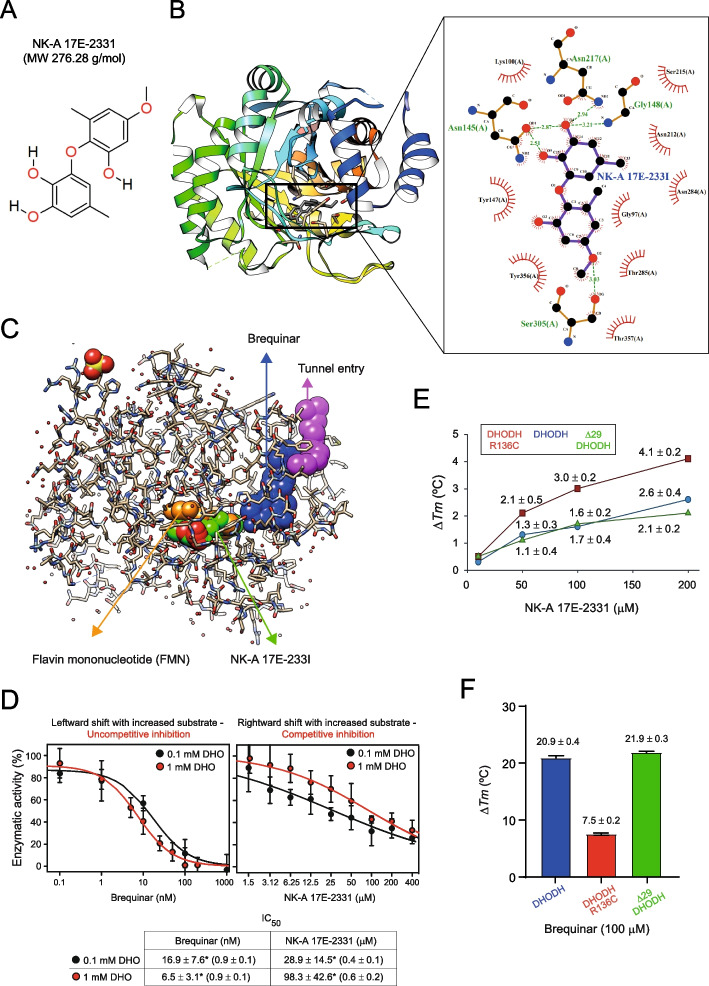
NK-A 17E-233I as a Novel Competitive Inhibitor of Human DHODH. **A** The molecular structure of NK-A 17E-233I is illustrated. **B** Molecular docking analysis indicates the formation of a complex between NK-A 17E-233I and the human DHODH protein (PDB: 1D3G). **C** A superposition of NK-A 17E-233I, FMN, and Brequinar when bound to human DHODH is presented, emphasizing the narrow tunnel that leads to the active site. **D** Graphical representations and a tabular summary illustrate the relative inhibitory effects of Brequinar and NK-A 17E-233I on the activity of DHODH at different concentrations of DHO in an enzymatic assay. The lines represent the fit of the equation *V* = *V*_max_/(1 + (*I*/IC_50_)^*h*^) for all normalized data. The reported IC_50_ values are the mean ± SD (n = 4–6), with the average of the fitted slopes ± SD provided in parentheses. Statistical analysis was conducted using an unpaired t-test. **E,F** Thermal stability assessments were performed via nanoDSF. Δ*T*_m_ reflects the difference in melting temperatures *T*_m_ of DHODH in the presence of NK-A 17E-233I (**E**) or Brequinar (**F**). The values of the differences in melting temperatures, Δ*T*_m_ (°C), are presented as mean ± SD (n = 3). The melting temperatures (*T*_m_) for DHODH, DHODH R136C, and Δ29 DHODH in the absence of a compound were 55.7 °C, 57.7 °C, and 51.5 °C, respectively

We discovered the polyketide small molecule NK-A 17E-233I (Fig. 1A; See methods) through a prospective virtual screening approach (Fig. S1A). It is derived from the ascomycetous fungus *Preussia typharum* and possesses promising drug-like and lead-like properties (Fig. S1B). Fungal polyketides (e.g., radicicol) are secondary metabolites manufactured by fungi and exhibit various in vitro anticancer activities [35].

### NK-A 17E-233I functions as a competitive inhibitor of human DHODH, with respect to the natural substrate dihydroorotate (DHO)

Molecular docking (Fig. 1B,C) suggested that NK-A 17E-233I competes fully or partially with the DHODH natural substrate, DHO. LigPlot^+^ docking analysis revealed that NK-A 17E-233I binds to human DHODH protein (PDB: 1D3G) [5] via 5 hydrogen bonds with Asn145, Gly148, Asn217, and Ser305 residues (Fig. 1B). Asn145 and Gly148 are critical in interactions with FMN and orotate, as indicated by the crystal structures of human DHODH in complex with Brequinar (a classical inhibitor of DHODH) [5, 36]. Both Asn145 and Asn217 are essential for DHO binding where Asn217 is located within the active site loop. Ser305 is also responsible for FMN binding [36].

Human DHODH enzyme comprises N-terminal and C-terminal domains connected with an extended loop. It features a proximal redox site where FMN oxidizes DHO to orotate, and a distal site where ubiquinone oxidizes dihydro FMN (FMNH_2_) to FMN [5]. The N-terminal domain institutes the entrance of a hydrophobic tunnel leading into the FMNH_2_–ubiquinone redox site at the C-terminal domain. This tunnel is recognized as the binding site for traditional inhibitors of DHODH, such as Brequinar and Teriflunomide [5].

Different from Brequinar, our molecular docking data suggested that NK-A 17E-233I binds overlapping with orotate and FMN, meaning it occupies the active site for the substrate DHO. Consequently, we anticipated that NK-A 17E-233I would compete with the substrate DHO.

Therefore, we tested the inhibitory effect of NK-A 17E-233I in a DHODH enzyme activity assay at two different concentrations of DHO, specifically 0.1 mM and 1 mM. We determined the IC_50_ values of NK-A 17E-233I in comparison to Brequinar, which is regarded as one of the best-characterized inhibitors of human DHODH [5, 7]. IC_50_ values depend on assay conditions and, for competitive inhibitors, increase as the concentration of substrate increases. Higher concentrations of the substrate compete with the inhibitor for enzyme binding, requiring more inhibitor to attain an equivalent level of inhibition. However, uncompetitive inhibitors typically exhibit decreasing IC_50_ values as the concentrations of the substrate increase [37].

For a compound that is fully or partially competitive towards DHO, we anticipated an increase in the IC_50_ value with a rising concentration of the substrate, DHO, in the assay. Indeed, our observations revealed a 3.4-fold increase in the IC_50_ value of NK-A 17E-233I with increasing concentrations of DHO, especially when compared to Brequinar (Fig. 1D).

In parallel, we tested Brequinar. Brequinar, Teriflunomide (the active metabolite of Leflunomide and previously named A77 1726), and Atovaquone are reversible inhibitors known to bind in the hydrophobic tunnel [5–7, 36]. Teriflunomide and Atovaquone were described as uncompetitive inhibitors of human DHODH with respect to the substrate, DHO [6, 7, 38]. It is therefore no surprise that in our experiments, the IC_50_ value of Brequinar decreased with increasing the concentration of DHO (Fig. 1D). This IC_50_ value of Brequinar in the low nanomolar range is consistent with previous reports [10, 29].

From this series of experiments, we conclude that our data corroborate the hypothesis proposed by molecular docking that NK-A 17E-233I functions as a competitive inhibitor of human DHODH with respect to DHO. For a pure competitive inhibitor, utilizing the Michaelis–Menten constant (*K*_m_) values of DHODH from [28], the IC_50_ values presented in Fig. 1D could be employed to calculate the inhibitory constant (*K*_i_) for NK-A 17E-233I [37]. Our findings demonstrated that the *K*_i_ value of NK-A 17E-233I is 1.5 µM or 3.9 µM, derived from the IC_50_ values at 1 mM or 0.1 mM DHO, respectively. By comparison, the reported *K*_i_ of approximately 1 µM for Teriflunomide—a clinically approved DHODH inhibitor—underscores the comparable potency of NK-A 17E-233I [6].

Our experiments also indicated that the mode of action of NK-A 17E-233I differs from traditional inhibitors of DHODH that bind within the hydrophobic tunnel.

As an orthogonal technique, we examined the interaction between NK-A 17E-233I and Brequinar with recombinant human DHODH in a thermal stability assay (TSA). Brequinar is known to significantly stabilize human DHODH, and the values of the difference in the melting temperatures (Δ*T*_m_) presented in Fig. 1E,F corroborate this finding—consistent with [28]. In contrast, NK-A 17E-233I exhibited considerably less stabilization, albeit within a range consistent with DHO. For instance, in the same experimental setup [39], a concentration of DHO at 100 µM demonstrated a Δ*T*_*m*_ value of 3.5 °C, suggesting that NK-A 17E-233I stabilizes DHODH in a manner analogous to the substrate, DHO.

Furthermore, we conducted a comparative analysis of NK-A 17E-233I and Brequinar. We performed enzymatic and biophysical characterizations using two human DHODH variants to elucidate critical aspects of inhibitors of DHODH. The localization of DHODH relative to the micelles during the assay affects its interaction with both substrates and inhibitors [39]. The positioning of DHODH is governed by a transmembrane domain that anchors the enzyme to the outer surface of the inner mitochondrial membrane in vivo [4, 40].

Consequently, we replicated the experiments using an N-terminal truncated form of human DHODH (Δ29), where the N-terminal transmembrane domain was removed. Additionally, we utilized a variant of DHODH containing the R136C mutation. The R136 residue is pivotal for the interaction between DHODH and Brequinar, as well as other inhibitors that bind within the tunnel leading to the FMN cofactor, utilized by the DHODH substrate, ubiquinone [5]. Both Brequinar and NK-A 17E-233I exhibited similar IC_50_ values and thermal stabilization concerning DHODH, irrespective of the presence of its transmembrane domain (Fig. 1D–F and Table 1).

**Table 1:** The half maximal inhibitory concentration (IC_50_) for Brequinar and NK-A 17E-233I was determined using 1 mM dihydroorotate (DHO) as the substrate. The reported IC_50_ values are expressed as mean ± standard deviation (SD) (n = 3). The average of the fitted slopes ± SD is provided in parentheses

	IC_50_	
Enzyme	Brequinar (nM)	NK-A 17E-233I (µM)
DHODH R136C	no inhibitory effect	48.6 ± 30.8 (0.6 ± 0.2)
Δ29 DHODH	6.6 ± 5.7 (1.1 ± 0.2)	99.1 ± 47.8 (1 ± 0.8)

As previously reported in [28], Brequinar did not inhibit the enzymatic activity of the DHODH variant R136C within the concentration range tested here (Table 1). Furthermore, the extent of thermal stabilization of this variant by Brequinar varied significantly. In contrast, NK-A 17E-233I did not demonstrate a statistically significant reduction in the IC_50_ value for the inhibition of the DHODH variant R136C. NK-A 17E-233I also stabilized the variant R136C more than the wild-type DHODH (Fig. 1E,F). Unlike Brequinar, NK-A 17E-233I maintained binding activity against the variant R136C, indicating a distinct mode of interaction that does not depend on the conventional hydrophobic tunnel.

### NK-A 17E-233I exhibits cytotoxic effects in cancer cells

**Fig. 2:**
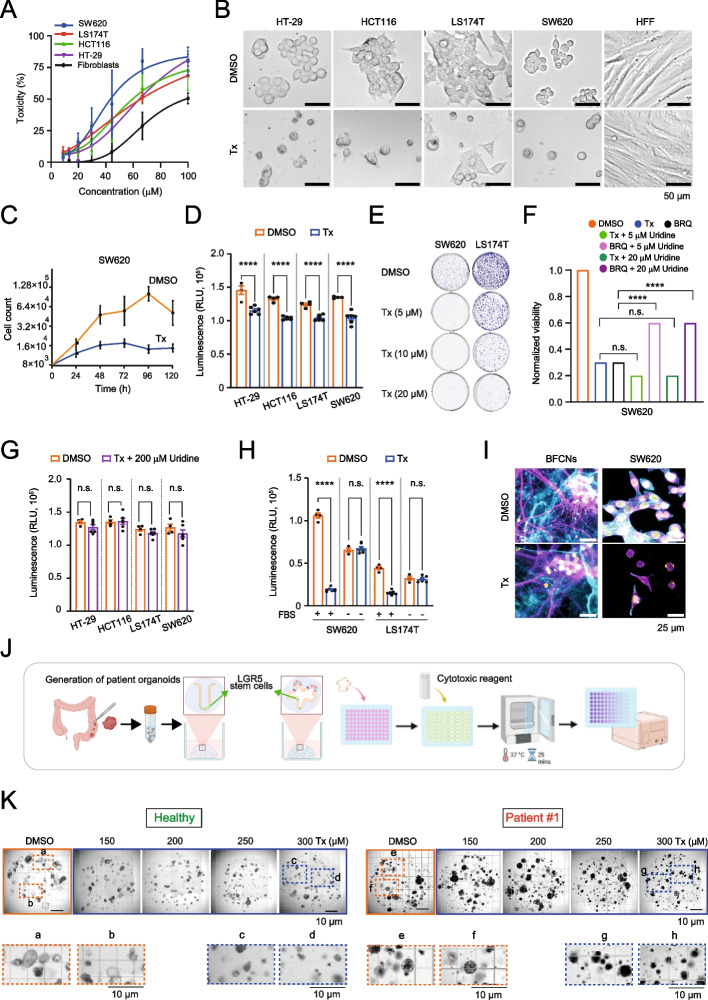
NK-A 17E-233I Demonstrates Cytotoxic Effects on Human Colorectal Cancer Cells and Patient-Derived Organoids. **A** Dose–response curves illustrate the cytotoxic effects of NK-A 17E-233I on human colorectal cancer cell lines and fibroblasts following a 48-h treatment period. Data are presented as mean ± SD (n = 3). **B** Representative brightfield images obtained using a Dmi8 Leica wide-field microscope depict the treatment effects (Tx, 25 µM) after a 48-h period. Scale bar = 50 µm. **C,D** The viability of colorectal cancer cells was assessed over time in the absence (DMSO) or presence of treatment (Tx, 25 µM), by the Trypan Blue exclusion assay (**C**) and the ATP assay over a 24-h treatment period (**D**).** E** A clonogenic assay provides representative images of colorectal cancer colonies following treatment (Tx, 25 µM) over a 14-day period at the specified concentrations. **F** The proliferation of colorectal cancer cells was assessed after 24 h as described in **C**, both in the absence (DMSO) and in the presence of Tx (25 µM) or BRQ (100 µM), along with physiological concentrations of uridine.** G** Similar to **D,** this portion assesses the outcomes following DMSO or treatment (Tx, 25 µM) and the exposure to non-physiological uridine (200 μM) over a 24-h treatment period. **H** This figure parallels **D**, but colorectal cancer cells were cultured in the absence or presence of FBS (10%) concurrently with DMSO or Tx (25 µM). Measurements (in relative light unit, RLU) in **D**, **G,** and** H** were obtained via the CellTiter Glo assay. Data are reported as mean ± SEM (n = 4–6). Statistical analyses were conducted using a one-way ANOVA test with Sidak’s multiple comparisons test. **I** Representative immunofluorescence images illustrate the morphology of BFCNs and SW620 cells following treatment (Tx, 25 µM) for 24 h, highlighting F-actin (in magenta), MAP2 (neurite marker, in cyan), and nuclei (DAPI, in yellow). Scale bar = 25 μm. **J** A schematic representation outlines the generation of organoids from colorectal cancer patients. **K** Representative images depict the morphology of healthy and patient-derived intestinal organoids subjected to increasing concentrations of Tx or DMSO for 96 h. Insets, delineated by dashed boxes in the top panel, emphasize the morphological features of the organoids. Scale bar = 10 µm. Tx and BRQ denote NK-A 17E-233I and Brequinar, respectively

Together with the in vitro biochemical assays described above, we investigated the cytotoxicity of NK-A 17E-233I in cancer cells. NK-A 17E-233I reduced the viability, cell proliferation, and the levels of adenosine triphosphate (ATP) in a dose-dependent manner across various human colorectal cancer cell lines (Fig. 2A-D and Fig. S2A). Colorectal cancer cells displayed altered morphology, characterized by a more rounded appearance, highlighting detachment. Furthermore, NK-A 17E-233I significantly diminished the ability of colorectal cancer cells to form colonies in clonogenic assays after two weeks (Fig. 2E). NK-A 17E-233I also showed cytotoxic effects in cell lines from other cancer types, including osteosarcoma, breast, and lung cancers (Fig. S2B). However, no similar effects were observed in healthy fibroblasts and keratinocytes (Fig. 2B and Fig. S2B).

In all assays, a concentration of 25 µM of NK-A 17E-233I was utilized. The IC_50_ value for Brequinar established at 100 µM was determined in SW620 cells and served as a reference point for comparative analyses in the subsequent experiments conducted in this study (Fig. S2C).

### NK-A 17E-233I maintains efficacy at physiological concentrations of uridine

Plasma uridine serves as an inherent resistance mechanism to the inhibition of DHODH in cancer therapy. For instance, cancer cells exhibit considerable resistance to Brequinar at physiological concentrations of uridine (5–20 µM) [41]. This prompted us to evaluate whether NK-A 17E-233I would continue to suppress cancer proliferation in the presence of 5 µM and 20 µM uridine in comparison to Brequinar. Our findings indicated that both concentrations of uridine did not mitigate the growth inhibitory effects of NK-A 17E-233I, unlike Brequinar (Fig. 2F). This distinctive property of NK-A 17E-233I would eliminate the necessity for complex combination strategies to obstruct salvage pathways.

The sustained efficacy of NK-A 17E-233I at physiological concentrations further suggests that it may exhibit improved intracellular retention (i.e., reduced efflux), contributing to prolonged target engagement and a lower likelihood of developing resistance. Indeed, our preliminary in silico data implied that NK-A 17E-233I is not a substrate of permeability glycoprotein (Fig. S1B). Permeability glycoprotein, a member of the ATP-binding cassette transporter family, functions as an efflux pump that facilitates the removal of anticancer drugs from cancer cells, thereby conferring drug resistance [42]. These observations suggest that NK-A 17E-233I is a promising candidate for further preclinical evaluation as a next-generation inhibitor of DHODH.

We further evaluated the effect of NK-A 17E-233I at a higher concentration of uridine (non-physiological, 200 µM) to determine the capacity of exogenous uridine to rescue pyrimidine synthesis via the salvage pathway. NK-A 17E-233I did not impact the production of ATP or cancer cell proliferation at various time points (Fig. 2G and Fig. S2D). At elevated concentrations of uridine, NK-A 17E-233I exhibited a loss of activity. This observation corroborates that its effects are attributable to the inhibition of pyrimidine de novo synthesis rather than off-target toxicity.

###  NK-A 17E-233I selectively spares quiescent cells, indicating targeted inhibition of pyrimidine de novo biosynthesis in cancer cells

To determine whether the anticancer activity of NK-A 17E-233I is mediated through the pyrimidine de novo synthesis pathway, we examined its effects on non-proliferative cells, which are expected to rely less on this pathway than tumor cells [2].

We treated non-cycling cancer cells (i.e., fetal bovine serum [FBS]-starved cancer cells and neurons), whose proliferative capacity is limited. As anticipated, NK-A 17E-233I had no significant effect on starved cancer cells (Fig. 2H), and basal forebrain cholinergic neurons (BFCNs) retained their dendritic and cytoskeletal structures, as evidenced by F-actin and the classic microtubule-associated protein 2 (MAP2) marker staining (Fig. 2I). However, it is important to note that serum starvation may also induce cellular stress responses (e.g., upregulation of autophagy), which could facilitate nucleoside salvage pathways and contribute to the availability of pyrimidine, irrespective of proliferation. While our results support the selectivity of NK-A 17E-233I for rapidly proliferating cells, they should be interpreted cautiously given the potential compensatory salvage mechanisms under starvation.

### NK-A 17E-233I displays anticancer activity in three-dimensional (3D) intestinal organoids

The cytotoxic effects of NK-A 17E-233I in colorectal cancer cells encouraged us to evaluate it in patient-derived 3D intestinal organoids. Organoids preserve the genetic, epigenetic, and phenotypic characteristics of the original patient tissue and serve as helpful preclinical platforms for predicting cancer drug response [43]. In contrast to those derived from healthy intestinal tissues, the tumor-derived organoids (Fig. 2J,K) demonstrated sensitivity to NK-A 17E-233I in a dose-dependent manner (Fig. S2E,F), reinforcing its effects observed in human cancer cell lines.

### NK-A 17E-233I modulates the transcriptomic profile of SW620 cancer cells

**Fig. 3:**
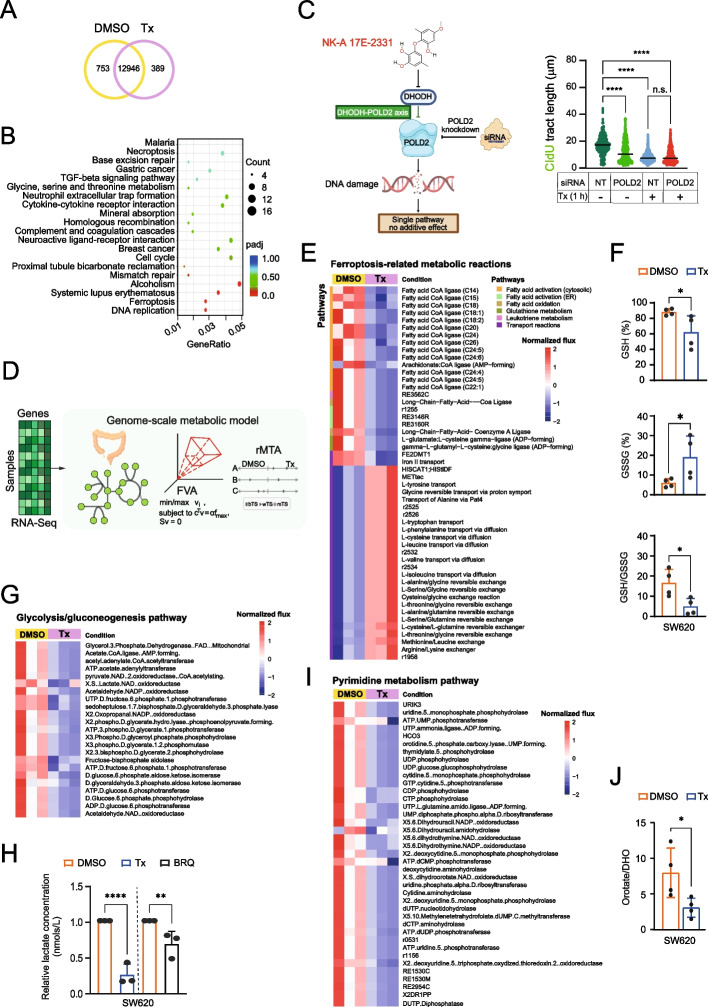
The Impact of NK-A 17E-233I on the Transcriptomic and Metabolomic Profile of SW620 Cells.** A** The Venn diagram illustrates the overlap and divergence of gene counts obtained from RNASeq following DMSO or treatment (Tx, 25 µM) for 48 h. **B** Kyoto Encyclopedia of Genes and Genomes (KEGG) enrichment scatter plot depicting the 20 most significant KEGG pathways, with the size of each point corresponding to the number of genes associated with each biological pathway. **C** Left: Schematic representation of the DHODH-POLD2 axis in cancer. Right: CldU tract lengths following treatment (Tx, 25 µM) for 1 h in knockdown cells. The median (n = 3) is indicated by a black line, with each dot representing a single fiber (≥ 200 fibers measured per condition). Statistical differences were determined using the Mann–Whitney test.** D** Construction of a sample-specific genome-scale metabolic model: A genome-scale metabolic model tailored to the specific sample was developed (see methods section for comprehensive details). **E, G,** and** I** Heatmaps depicting the metabolic alterations subsequent to treatment with DMSO, Tx (25 µM), or BRQ (100 µM). **F**, **H,** and **J** represent the functional measurements of glutathione, lactate, and orotate/DHO ratio, respectively. Data are expressed as mean ± SEM (n = 4–5). Statistical differences were assessed using a two-way ANOVA test with Sidak´s multiple comparisons test. Tx and BRQ denote NK-A 17E-233I and Brequinar, respectively

We conducted RNA sequencing (RNASeq) on SW620 colorectal cancer cells, which represent a metastatic drug-resistant model [44] to identify the differentially expressed genes following treatment with NK-A 17E-233I. Figure 3A illustrates the number of uniquely expressed genes in untreated versus treated group in a coexpression Venn diagram. Gene ontology enrichment analysis indicated significant enrichment of the pathways related to ferroptosis and DNA replication (Fig. 3B). These results align with recent studies linking *DHODH* with DNA replication- and ferroptosis-related genes in cancer [45, 46].

Particularly, RNASeq analysis identified DNA polymerase delta subunit 2 (POLD2) as a significantly downregulated gene among those associated with DNA replication following treatment with NK-A 17E-233I (see 10.5281/zenodo.14833856). DNA polymerase delta subunit 2 encodes the second subunit of the DNA polymerase delta–holoenzyme, which is critical for DNA replication and repair [47]. It is often overexpressed in aggressive tumors such as colorectal cancer and correlates with poor prognosis.

To date, there is no direct experimental evidence establishing a functional relationship between *POLD2* and *DHODH* in cancer. Only a single correlation study has identified *POLD2* as one of the top four genes co-expressed with *DHODH* in colorectal adenocarcinoma [45]. These observations prompted us to investigate whether a direct relationship would exist between the inhibition of DHODH and POLD2. Therefore, we hypothesized that NK-A 17E-233I may impair DNA replication by affecting DHODH, thus limiting the ability of POLD2 to facilitate efficient replication fork progression during nucleotide stress conditions (as depicted in Fig. 3C).

### NK-A 17E-233I reveals a functional DHODH–POLD2 axis in DNA replication stress in cancer

To evaluate our hypothesis, we initially silenced *POLD2* using small interfering RNA (siRNA), which led to a significant reduction in the speed of replication forks, thereby confirming its critical role in sustaining DNA synthesis (Fig. 3C and Fig. S3A,B). Treatment with NK-A 17E-233I alone also significantly diminished the speed of replication forks by depleting the pools of intracellular pyrimidines (Fig. 3C). Importantly, the concurrent silencing of *POLD2* and treatment with NK-A 17E-233I did not further decrease fork speed compared to NK-A 17E-233I alone (Fig. 3C).

The absence of an additive effect suggests that NK-A 17E-233I-induced replication stress is directly linked to the function of POLD2. These findings establish the activity of DHODH as a prerequisite for POLD2-dependent DNA synthesis in cancer [47, 48]. By maintaining nucleotide pools, DHODH supports efficient replication, making POLD2-rich tumors vulnerable. This indicates the potential for utilizing inhibitors of DHODH in combination with other inhibitors to target complementary pathways, thereby enhancing anticancer efficacy. Inhibitors such as those targeting Rad3-related kinase (ATR) or poly (ADP-ribose) polymerase (PARP), including Olaparib, serve as pertinent examples.

These findings were substantiated by reductions in the mRNA and protein expression levels of POLD1 and POLD2, as assessed by RT-qPCR and western blot assays following treatment with NK-A 17E-233I. However, we did not observe a change in the expression levels of DHODH (Fig. S3C,D). We also noticed that the downregulation of POLD2 modulated the expression of translesion synthesis (TLS) polymerase and mismatch repair genes (Fig. S3C). In the literature, POLD2 is linked to mismatch repair and TLS polymerase pathways, with its overexpression being correlated with drug resistance in cancer [47].

### NK-A 17E-233I triggers ferroptosis in cancer cells

We employed a metabolic modeling approach (Fig. 3D) based on our RNASeq data to investigate the core metabolic reactions driving ferroptosis, a regulated cell death pathway modulated by the inhibition of DHODH [46]. NK-A 17E-233I significantly downregulated the metabolism of glutathione (Fig. 3E), which is an essential defense mechanism against ferroptosis; its disruption sensitizes cancer cells to oxidative damage [49]. This effect impaired antioxidation, disrupted mitochondrial fatty acid β-oxidation, and diminished fatty acid biosynthesis, proved by the reduced activity of γ-L-glutamyl-L-cysteine:glycine ligase reactions.

The quantitative measurements of glutathione in SW620 cells corroborated our transcriptomic and metabolic findings (Fig. 3F). NK-A 17E-233I significantly decreased the levels of the reduced form (GSH) of glutathione and the GSH/GSSG ratio, while concurrently increasing the levels of its oxidized form (GSSG). Collectively, NK-A 17E-233I promoted ferroptosis by disrupting the redox balance and suppressing the essential metabolic pathways in cancer cells.

### NK-A 17E-233I diminishes the glycolytic activity of cancer cells

Glycolysis represents a hallmark of cancer, wherein tumor cells preferentially metabolize glucose anaerobically rather than aerobically [50]. The transcriptomics-based metabolic model indicated that NK-A 17E-233I downregulated the key enzymes involved in glycolysis (Fig. 3G). NK-A 17E-233I significantly reduced the concentration of lactate in comparison to Brequinar in SW620 cells (Fig. 3H). The simultaneous disruption of nucleotide synthesis and glycolysis by NK-A 17E-233I may limit metabolic adaptability and enhance the efficacy of cytotoxic therapies (e.g., chemotherapy).

###  NK-A 17E-233I impairs pyrimidine de novo biosynthesis by depleting orotate in cancer cells

We employed a condition-specific metabolic modeling approach to assess the impact of NK-A 17E-233I on pyrimidine metabolism in cancer cells. NK-A 17E-233I downregulated the (S)-dihydroorotate:NAD⁺ oxidoreductase reaction (kegg.jp/entry/R01869), catalyzed by DHODH to produce orotate (Fig. 3I). Consistently, flux analysis revealed reduced levels of orotate, citrate, and glutamine, which are key components of pyrimidine de novo synthesis (Fig. S3F,G). These findings align with previous studies reporting that the inhibition of DHODH reduces citrate and the availability of glutamine [51, 52].

Accordingly, we validated the on-target activity of NK-A 17E-233I on the pyrimidine de novo pathway, by quantifying the amounts of DHO, orotate, and nucleotides following treatment with NK-A 17E-233I in SW620 cells using HPLC–MS/MS. NK-A 17E-233I significantly decreased the orotate/DHO ratio (Fig. 3J and Table 2), indicating a partial inhibition of DHODH-mediated DHO oxidation to orotate. This enzymatic inhibition was further supported by a significant depletion in the uridine nucleotide pool (UMP + UDP + UTP), suggesting an impairment in the synthesis of uridine. In contrast, the levels of purine nucleotides remained unchanged. The data distribution for the cytidine pool, another pyrimidine, also displayed a downward trend, although data dispersion precluded statistical significance.

**Table 2:** The metabolite content analysis in total SW620 cell extract using HPLC–MS/MS. The relevant concentrations of metabolites were determined following a 24-h treatment with NK-A 17E-233I at a concentration of 25 µM. The results reported represent the median of five independent experiments (four for dihydroorotate [DHO] and orotate) with the range indicated as [Minimum – Maximum]

	**Metabolite amount (pmoles/mg protein)**	
	**DMSO**	**NK-A 17E-233I**
DHO	15.9 [1.5–39.7]	26.5 [18.3–228.5]
Orotate	126.2 [18.4–228.5]	70.5 [61.7–137.1]
**Orotate/DHO Ratio**	**7.3 [4.8** –** 12.4]**	**3.1 [1.5** –** 4.6]**
AMP	587 [252–1,263]	961 [405–2,739]
ADP	7,714 [3,725–8,617]	7,917 [5,048–9,389]
ATP	23,585 [15,053–26,136]	15,907 [14,173–21,694]
**AMP + ADP + ATP**	**30,929 [19,031** –** 35,336]**	**26,257 [24,352** –** 27,848]**
GMP	141 [0–345]	156 [0–268]
GDP	1,139 [679–1,228]	1,339 [1,008–1,566]
GTP	4,627 [3,688–6,690]	3,565 [3,407–6,418]
**GMP + GDP + GTP**	**5,731 [4,916** –** 8,173]**	**5,228 [4,902** –** 7,797]**
CMP	322 [182–1,029]	263 [220–707]
CDP	292 [142–591]	351 [143–504]
CTP	9,583 [5,797–14,709]	5,801 [5,142–9,519]
**CMP + CDP + CTP**	**10,197 [6,121** –** 16,281]**	**6,327 [6,028** –** 10,285]**
UMP	189 [66–453]	335 [81–424]
UDP	1,476 [827–1,954]	927 [867–1,161]
UTP	9,716 [6,288–11,240]	4,013 [3,397–6,651]
**UMP + UDP + UTP**	**11,858 [7,869** –** 13,647]**	**5,275 [4,713** –** 7,687]**

*Abbreviations*: *ADP *Adenosine diphosphate, *AMP *Adenosine monophosphate, *ATP *Adenosine triphosphate, *CDP *Cytidine diphosphate, *CMP *Cytidine monophosphate, *CTP *Cytidine triphosphate, *DHO *Dihydroorotate, *DMSO *Dimethyl sulfoxide, *GDP *Guanosine diphosphate, *GMP *Guanosine monophosphate, *GTP *Guanosine triphosphate, *UDP *Uridine diphosphate, *UMP *Uridine monophosphate, *UTP *Uridine triphosphate. Note: The levels of GMP were undetectable in certain extracts; for statistical analysis, these values were treated as zero

### NK-A 17E-233I disrupts mitochondrial membrane potential (ΔΨm) and the production of reactive oxygen species (ROS).

**Fig. 4:**
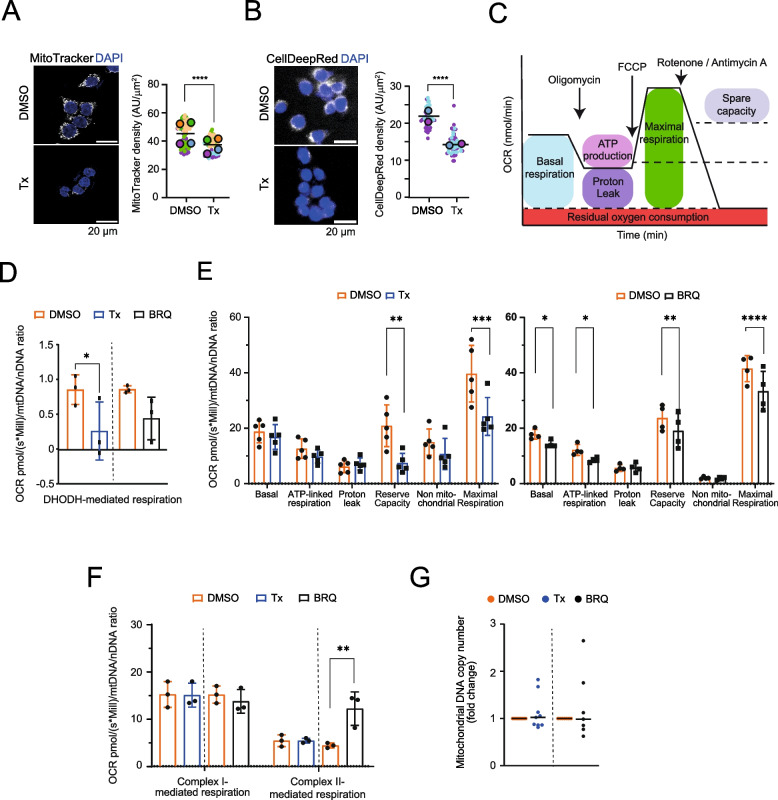
The Impact of NK-A 17E-233I on Mitochondrial Bioenergetics in Cancer Cells. **A,B** Representative confocal microscopy images demonstrate the effects of DMSO or Tx (25 µM) administered for 24 h on HCT116 colorectal cancer cells, with a focus on mitochondrial activity assessed using MitoTracker Red (grey) to visualize the mitochondrial membrane potential (**A**) or ROS production (**B**) evaluated with CellDeepRed staining (grey). Nuclei were stained with DAPI (blue). Scale bar = 20 µm. Violin plots depict the density of MitoTracker (**A**) or CellDeepRed (**B**) density per cell, calculated as the intensity of MitoTracker or ROS divided by the area of each cell. N represents independent replicates (2–4) for each condition; n denotes the density of MitoTracker or ROS per cell. In** A,B**, the data are presented as ‘Superplots,’ indicating the mean of the various replicates (large circles) alongside the distribution of ‘MitoTracker or ROS density per cell analyzed’ (color-coded dots), which is superimposed as a beeswarm plot. The black line represents the mean, and a paired two-tailed t-test was utilized to assess the statistical differences. **C** A diagram illustrating the oxygen consumption rate (OCR) profile delineates the key phases of mitochondrial respiration. **D-F** Analysis of OCR in colorectal cancer cells following a 24-h treatment with DMSO, Tx (25 µM) or BRQ (100 µM). **D** DHODH-mediated respiration; **E** The measurements of OCR under basal conditions and subsequent to the sequential administration of inhibitors of OXPHOS. **F** Complex I- and complex II-mediated respiration. Data from **D-F** are expressed as mean ± SEM (n = 3–5). Statistical differences were determined using a two-way ANOVA test with Sidak’s multiple comparisons test. **G** Mitochondrial DNA copy number was quantified via qPCR at the specified conditions. Tx and BRQ denote NK-A 17E-233I and Brequinar, respectively

Dihydroorotate dehydrogenase plays a crucial role in maintaining ΔΨm, with its reduction serving as a critical marker of mitochondrial dysfunction. The oxidation of DHO to orotate may also influence the production of ROS [52–56]. NK-A 17E-233I significantly decreased both ΔΨm and ROS levels (Fig. 4A,B) in cancer cells, consistent with findings from other inhibitors of DHODH [57]. For example, Teriflunomide has been shown to diminish the levels of mitochondrial ROS and induce cell death in cancer cells [58]. However, another study indicated that the inhibition of DHODH increases the generation of ROS in cancer cells [54]. Furthermore, in different cancer cell lines, NK-A 17E-233I decreased ΔΨm without significant effect on the levels of ROS (Fig. S4A,B). The relationship between DHODH and the production of ROS in cancer remains controversial and warrants further investigation.

### NK-A 17E-233I does not exhibit off-target effects on mitochondrial complexes I and II

The DHODH enzyme is located on the outside of the inner mitochondrial membrane, and its activity depends on ubiquinone as an electron acceptor [2]. Upon the reduction of ubiquinone by DHODH, ubiquinone diffuses into the internal mitochondrial membrane to be reoxidized through complex III- and complex IV-mediated respiration [59]. The direct contribution of DHODH to routine mitochondrial oxygen consumption is minimal; DHODH knock-out cells demonstrated normal bioenergetics, ROS production, and oxidative phosphorylation (OXPHOS)-derived ATP levels [60].

Initially, we confirmed that NK-A 17E-233I specifically affects DHODH-mediated oxygen consumption in the presence of excess exogenous DHO in digitonin-permeabilized SW620 cells. NK-A 17E-233I and Brequinar reduced the oxygen consumption rate (OCR) in the presence of exogenous DHODH substrates, although the reduction was more pronounced in NK-A 17E-233I-treated cells (Fig. 4C,D). However, when we assessed various parameters of mitochondrial respiration in intact cells (i.e., mitochondrial respiration supported by endogenous substrates), NK-A 17E-233I only significantly decreased maximal respiration. In contrast, Brequinar decreased most of these respiration parameters (Fig. 4E). NK-A 17E-233I selectively impaired maximal mitochondrial respiration without affecting routine function, highlighting the pivotal role of DHODH role under high-energy demand conditions. The effect of NK-A 17E-233I was only observed when pushing the maximal capacity of OXPHOS. These findings also align with the understanding that DHODH contributes minimally (5–10%) to routine mitochondrial oxygen consumption [61].

Traditional inhibitors of DHODH (e.g., Brequinar) are recognized to significantly impact the OXPHOS system due to off-target effects on the complexes of the electron transport chain (ETC) [61]. We measured complex I- and complex II-mediated respiration rates in digitonin-permeabilized SW620 cells. NK-A 17E-233I did not alter the OCR supported by the presence of complex I and II substrates, whereas Brequinar stimulated complex II-mediated respiration (Fig. 4F).

Overall, the observed effects on mitochondrial respiration here were not attributed to changes in mitochondrial DNA copy number (Fig. 4G).

Given that Brequinar significantly decreased basal and ATP-linked respiration in intact cells, the increase in complex II-mediated respiration may reflect a compensatory mechanism to partially restore OXPHOS activity. These findings suggest that NK-A 17E-233I is a potent inhibitor of DHODH with no off-target effects on the ETC compared to conventional inhibitors.

### NK-A 17E-233I causes fork stalling, DNA breaks, and checkpoint activation in cancer cells

**Fig. 5:**
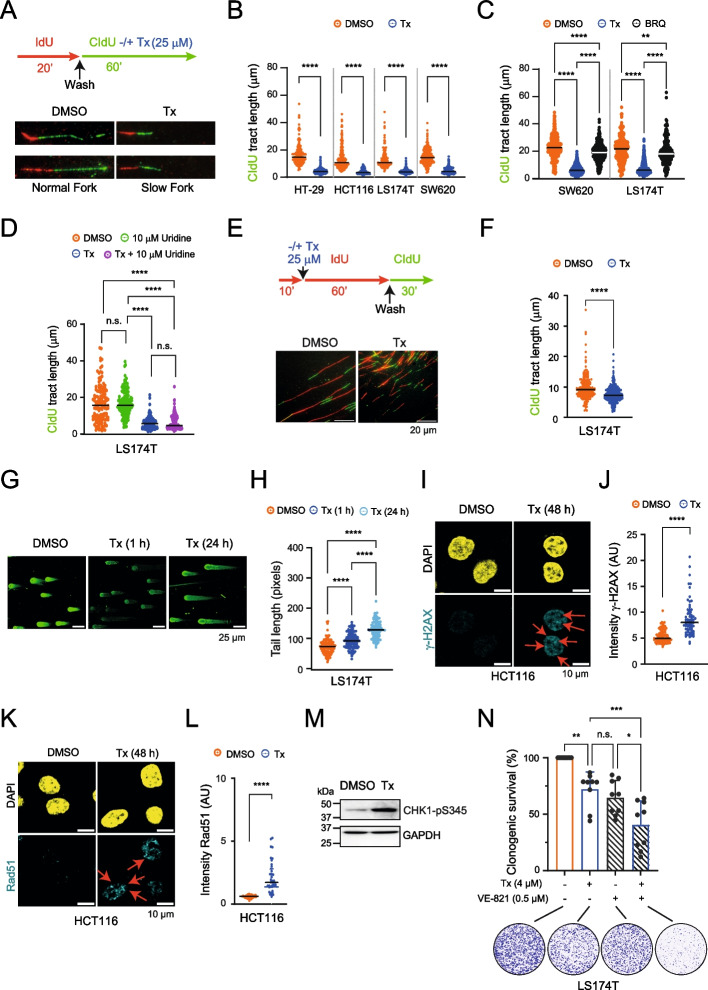
The Impact of NK-A 17E-233I on Fork Progression and Induction of DNA Damage in Colorectal Cancer Cells. Cells were treated with DMSO, Tx (25 µM), or BRQ (100 µM) for a 1-h period. **A** Top: Schematic representation of the IdU/CldU pulse-labeling protocol. Bottom: Representative images of DNA fibers. **B-D** CldU tract lengths at the specified conditions (n = 3). Each dot represents one fiber (≥ 200 fibers were measured per condition). **E** Top: Schematic representation of the IdU/CldU pulse-labeling protocol to evaluate fork restart. Bottom: Representative images of DNA fibers following fork restart in LS174T cells at the specified conditions. Scale bar = 20 μm. **F** CldU tract lengths in LS174T cells from **E.** Each dot represents one fiber (> 140 fibers were measured per condition), with the horizontal line representing the median from one experimental replicate (n = 3). **G** Representative fluorescence images demonstrating the staining of DNA breaks in single-cell neutral gel electrophoresis (neutral comet assay). Scale bar = 25 μm.** H** Quantification of the DNA comet tail length from one experimental replicate. Each dot represents one cell (> 95 cells were measured per condition) (n = 3). **I** Representative immunofluorescence images displaying staining of the DNA damage marker protein γ-H2AX (cyan) and the nucleus (DAPI, yellow). Red arrows indicate DNA damage in treated cells. **J** Violin plots illustrating the fluorescence intensity of the nuclear γ-H2AX (n = 3). Each dot represents one cellular nucleus (n ≥ 74 nuclei were measured per condition). **K** as in **I**, but displaying staining of Rad51 (cyan).** L** as in **J**, but derived from Rad51 images from **K**. **M** Western blot analysis showing CHK1-pS345 and GAPDH (loading control). **N** Top: Percentage of colonies of LS174T cells after treatment with DMSO, Tx (4 µM), VE-821 (0.5 µM), or the combination of both. Bars represent the mean ± SD (n = 9), shown as dots. Bottom: A representative image of human colorectal cancer colonies conducted at the specified conditions for 14 days. Statistical differences were determined using the Mann–Whitney test in all panels, except in **H** and** N** where a one-way ANOVA test with Bonferroni’s multiple comparisons test was employed. Unless otherwise specified, all results are expressed as the median (horizontal black line). Tx and BRQ denote NK-A 17E-233I and Brequinar, respectively

We examined the effects of NK-A 17E-233I on the progression of replication forks in a panel of colorectal cancer cell lines utilizing DNA fiber assays, as pyrimidine insufficiency is known to disrupt the dynamics of replication forks [62]. The dynamics of replication forks can be assessed through the incorporation of fluorescently-labeled nucleoside analogues into the newly synthesized DNA strands (Fig. 5A).

Treatment with NK-A 17E-233I significantly reduced the speed of replication forks, as indicated by shorter tracks of incorporated 5-Chloro-2'-deoxyuridine (CldU) compared to untreated controls (Fig. 5 A,B). The effect of NK-A 17E-233I is significant when compared to Brequinar (Fig. 5C). Furthermore, physiological concentrations of uridine (10 µM) did not demonstrate any discernible rescue in the speed of replication forks by NK-A 17E-233I (Fig. 5D). This supports our earlier data that NK-A 17E-233I retained its activity at physiological concentrations of uridine (Fig. 2F). Excess concentrations of uridine (200 µM) rescued the DNA-damaging effects of NK-A 17E-233I (Fig. S5A), highlighting its on-target activity on the pyrimidine de novo pathway as shown in Fig. 2G and Fig. S2D.

Following the washout period, replication forks continued to demonstrate a diminished progression rate, indicating the prolonged cellular effects of NK-A 17E-233I (Fig. 5E,F and Fig. S5B). Additionally, a neutral comet assay was performed to detect and quantify DNA damage at the single-cell level resulting from the depletion of pyrimidines [20, 63]. Cells treated with NK-A 17E-233I displayed longer comet tails with increased DNA intensity compared to untreated controls, indicating the accumulation of fragmented DNA due to double-strand breaks (Fig. 5G,H). That DNA damage was further corroborated by immunofluorescence experiments, which revealed a significant increase in the intensity of the γ-H2AX and Rad51 DNA damage response proteins (Fig. 5I-L). These findings suggest that colorectal cancer cells likely activate DNA repair mechanisms to mitigate replication stress after treatment with NK-A 17E-233I.

The inhibition of DHODH induces replication stress, prompting cancer cells to activate the ATR/checkpoint kinase 1 (CHK1) pathways for DNA repair [64]. Western blot analysis confirmed the phosphorylation of CHK1 following treatment with NK-A 17E-233I compared to untreated controls (Fig. 5M and Fig. S5C). In clonogenic assays, NK-A 17E-233I exhibited synergistic activity in combination with the ATR inhibitor VE-821 (Fig. 5N and Fig. S5D). The inhibition of ATR disrupts key replication stress checkpoints, causing aberrant cell cycle progression and increased replication stress that culminates in cancer cell death. NK-A 17E-233I potentiates the inhibition of ATR to enhance anticancer efficacy, supporting our earlier assumptions that dual DHODH/ATR targeting intensifies replication stress and tumor cell death.

Cancer cells are likely to be incapable of resuming their cell cycle and may undergo apoptosis due to the accumulation of fragmented DNA.

### NK-A 17E-233I inhibits the progression of cell cycle in cancer cells

**Fig. 6:**
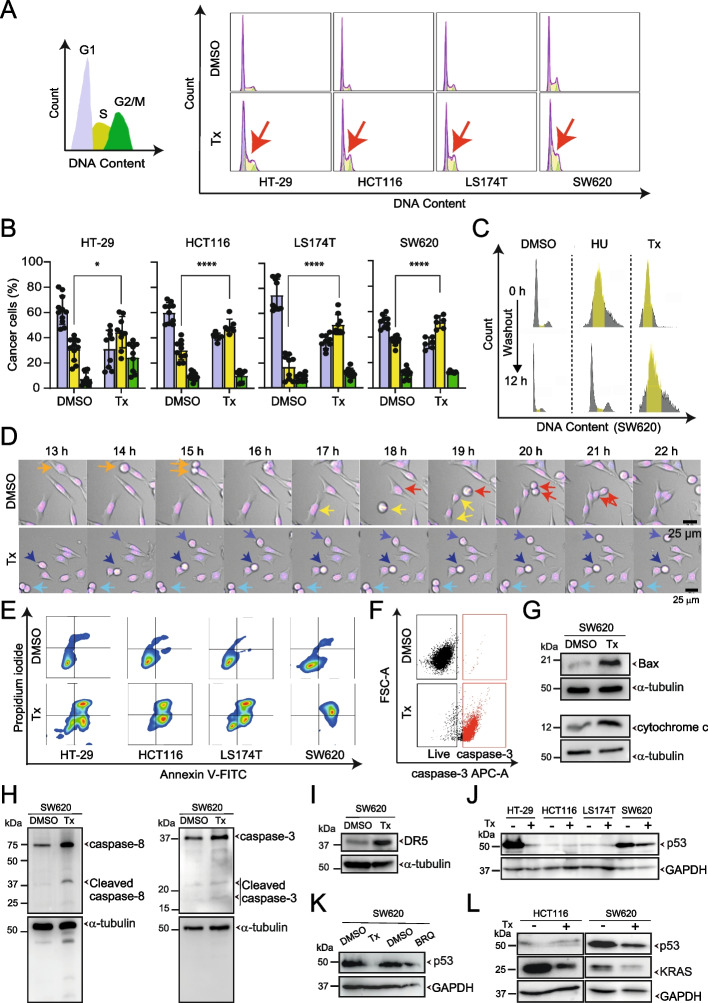
NK-A 17E-233I Inhibits the Progression of Cell Cycle and Induces Apoptosis in Colorectal Cancer Cells. **A** The left panel depicts a flow cytometry schematic that illustrates the distribution of DNA content across the G1, S, and G2/M phases of the cell cycle. The right panel features a representative FlowJo histogram displaying the DNA content in a cohort of colorectal cancer cells (n = 3). The red arrow indicates the accumulation of cancer cells in the S phase following a 48-h treatment period with DMSO or Tx (25 µM). **B** The proportion of cancer cells within each phase of the cell cycle is presented, with data expressed as mean ± SD (n ≥ 6). **C** Flow cytometry analysis conducted using CytExpert reveals the DNA content of colorectal cancer cells. The upper section demonstrates the population of cells arrested following treatment with DMSO, hydroxyurea (HU, 1 mM), or Tx (25 µM), while the lower section presents the washout results after 12 h, comparing Tx-released cells to those treated with HU. A representative image is provided (n = 3), with yellow sections indicating the cells arrested in the S-phase. **D** Representative microscopy images from time-lapse experiments display brightfield images (cell morphology in grey) alongside nuclei stained with Hoechst 33,342 (magenta). The arrows indicate the cells approaching division and/or in the process of division, demonstrating that treated cells exhibit a prolonged duration prior to division compared to untreated cells. Scale bar = 25 μm. **E** Representative dot plots from the Annexin V-FITC assay following FACS analysis of colorectal cancer cells with DMSO or Tx (25 µM). **F** A representative dot plot from FACS analysis illustrates the activation of caspase-3 in colorectal cancer cells in the presence of DMSO or Tx (25 µM). The black boxes denote live cells, while the red ones indicate dead cells. **G-L** Representative western blots depict the expression levels of Bax, cytochrome c, caspase-8, and caspase-3 (including both non-cleaved and cleaved protein products), as well as DR5, p53, and KRAS protein levels in untreated versus treated cells (n = 3). α-tubulin and GAPDH served as loading controls. Tx and BRQ denote NK-A 17E-233I and Brequinar, respectively

Treatment with NK-A 17E-233I (25 µM) resulted in the arrest of colorectal cancer cells in the S-phase and an increase in sub-G1 cell populations, as demonstrated by flow cytometry (Fig. 6A,B and Fig. S6A). When compared to hydroxyurea (HU, 1 mM), a known S-phase blocker, NK-A 17E-233I resulted in a similar initial cell cycle arrest. Following washout, only HU-treated cells resumed proliferation, whereas NK-A 17E-233I-treated cells remained arrested and/or underwent cell death (Fig. 6C). Live-cell imaging revealed that, unlike HU-treated cells, those exposed to NK-A 17E-233I failed to progress through mitosis (G2/M), became fragmented, and displayed morphological characteristics of apoptosis (Fig. 6D and Fig. S6B).

### Beyond ferroptosis, NK-A 17E-233I induces apoptosis

Dihydroorotate dehydrogenase also inhibits apoptosis by preserving ΔΨm in cancer cells [2]. Annexin V-FITC and active caspase-3 staining demonstrated that NK-A 17E-233I significantly increased the proportion of late-apoptotic colorectal cancer cells (Fig. 6E,F). We also confirmed that the effects of NK-A 17E-233I are caspase-dependent, as co-treatment with the pan-caspase inhibitor Z-VAD-FMK (10 µM) abolished its activity (Fig. S6C).

NK-A 17E-233I appears to activate both mitochondrial and death receptor-mediated apoptosis pathways. Western blot analysis confirmed increased levels of key apoptotic markers, including TRAIL receptor 2 (DR5), caspase-8, Bax, cytochrome c, and caspase-3 (Fig. 6G-I and Fig. S6D,E). The upregulation of DR5 suggests a potential sensitization to TRAIL-mediated apoptosis, possibly through paracrine signaling in response to DNA damage.

Although the activation of TRAIL pathway was not functionally tested in this study, these findings align with previous reports linking the inhibition of DHODH to TRAIL signaling [65]. However, the upregulation of DR5 alone does not establish causality. Hence, further investigations using genetic or pharmacological approaches (e.g., DR5/DR3 knockdown or TRAIL co-treatment) are necessary to elucidate its role in NK-A 17E-233I-induced cell death.

### NK-A 17E-233I modulates apoptosis-related protein expression in p53- and KRAS-mutant tumors

The mutations in the tumor suppressor p53 are a prevalent mechanism through which cancers evade apoptosis and resist genotoxic conditions [50]. NK-A 17E-233I reduced the levels of p53-mutant protein without influencing the expression of the wild-type protein in colorectal cancer cell lines (Fig. 6J and Fig. S6F). This reduction may contribute to the suppression of glycolytic flux observed earlier in this study (Fig. 3G,H), consistent with the established role of p53-mutant protein in promoting glycolysis [66]. Brequinar, in contrast, did not have an observed effect (Fig. 6K and Fig. S6G).

Given the co-occurrence of KRAS mutations in colorectal cancer [67], we also evaluated the impact of NK-A 17E-233I on KRAS-mutant proteins. NK-A 17E-233I reduced the expression of the KRAS glycine-to-aspartic acid mutant protein at codon 13 (KRAS^G13D^) in HCT116 cells and the KRAS glycine-to-valine homozygous mutation at codon 12 (KRAS^G12V^) in SW620 cells—which harbor p53- mutant protein (Fig. 6L and Fig. S6H). These KRAS variants constitute two of the most prevalent oncogenic mutations in colorectal cancer [67]. This remark aligns with another study indicating the synthetic lethality of Brequinar in KRAS-mutant cells [52].

## Discussion

We introduced NK-A 17E-233I as a novel competitive inhibitor of human DHODH, specifically targeting the enzyme’s natural substrate, DHO. This contrasts with the traditional approaches targeting the hydrophobic tunnel leading to the FMN binding cavity. Molecular docking and biochemical analyses indicate that NK-A 17E-233I functions as either a pure or partial competitive inhibitor of DHODH. NK-A 17E-233I emerges as a selective inhibitor of DHODH that preserves mitochondrial respiration through complex I and II substrates and maintains ATP-linked basal respiration—distinguishing itself from all traditional inhibitors, which are associated with an impairment of the ETC and hepatotoxicity [68–70].

Overall, NK-A 17E-233I stands out for its biochemical profile among other inhibitors used in clinical settings or clinical trials of the past three decades. Only two compounds, 3,4- and 3,5-dihydroxybenzoate, have been shown to occupy the DHO site in *Lactococcus lactis* DHODH, though their activity is species-specific [71]. A few other compounds were also defined as weak substrate-based inhibitors of DHODH with respect to DHO, such as 5-aza-DHO, 5-methyl orotic acid, and d,l -5-*trans*-methyl DHO on rat DHODH [72]. Additional compounds tested on non-mammalian DHODHs are listed in [73]. However, the precise determination of its *K*_*i*_ and the acquisition of an X-ray co-crystal structure of the NK-A 17E-233I–DHODH complex are imperative for a comprehensive understanding of its mechanism of binding.

Although the IC_50_ value of NK-A 17E-233I was higher than that of Brequinar in biochemical assays, the IC_50_ values are assay-dependent and mode of inhibition-dependent. The scale is evident when we compare NK-A 17E-233I to orotate, which is a DHODH reaction product and competitive inhibitor concerning DHO (*K*_i_ = 11 µM for human DHODH) [74]. The IC_50_ values that we reported , along with the assumption that NK-A 17E-233I functions as a pure competitive inhibitor towards DHO, imply *K*_i_ values of 1.5 µM or 3.9 µM. This places NK-A 17E-233I as a novel inhibitor of DHODH in the low micromolar range with a higher affinity than orotate.

Physiological uridine bypasses the blockade of pyrimidine de novo synthesis, significantly restraining the effectiveness of inhibitors of human DHODH [41]. For instance, physiological concentrations of uridine compromised the anticancer activity of the inhibitor of human DHODH, GSK983. This effect was mitigated by the coadministration of dipyridamole, which blocks nucleoside transport, including uridine itself [41]. In the present study, NK-A 17E-233I retained its potency under physiological concentrations of uridine compared to conventional inhibitors of DHODH.

NK-A 17E-233I sustained antiproliferative and DNA-damaging activities across a range of uridine concentrations (5–20 µM) that span the levels of physiological plasma, in contrast to Brequinar. This underscores the potential of NK-A 17E-233I to overcome salvage pathway–mediated resistance, eliminating the need for transporter inhibition (e.g., Equilibrative Nucleoside Transporters, ENTs) or combination regimens that often increase toxicity.

Dihydroorotate dehydrogenase represents a pivotal intersection between pyrimidine de novo biosynthesis and mitochondrial respiration, facilitating electron transfer to ubiquinone within the ETC. Ubiquinone acts as a mediator for electrons derived from both complex I and complex II to complex III, thereby establishing a functional linkage between the metabolism of pyrimidine and the mitochondrial ETC. The direct contribution of DHODH to routine mitochondrial oxygen consumption is marginal. This is supported by the fact that DHODH knock-out cells feature normal bioenergetics, ROS production, and OXPHOS-derived ATP normal levels. However, traditional inhibitors of DHODH impact substantially the OXPHOS system, at least in part, this has been attributed to their off-target effects in a DHODH-unrelated manner [61]. For instance, Brequinar possesses the potential to disrupt mitochondrial redox homeostasis by modifying electron flow within the ETC [61].

NK-A 17E-233I sustained mitochondrial respiration through complexes I and II, whereas Brequinar specifically stimulated the activity of complex II in cancer cells. This finding contrasts with previous reports that characterized Brequinar as an inhibitor of complex II associated with mitochondrial dysfunction [68–70]. This paradox (i.e., Brequinar increasing the activity of complex II) may reflect a compensatory mitochondrial adaptation in response to the inhibition of DHODH. While the upregulation of complex II may temporarily sustain redox balance, prolonged activation could lead to the accumulation of ROS and metabolic stress. Further investigations are necessary to elucidate these adaptive responses and their implications for cancer metabolism and drug-induced toxicity.

The preservation of the activity of complex II (and in part of complex I) by NK-A 17E-233I may correlate with a potential reduction in mitochondrial stress markers, including elevated ROS production, succinate accumulation, and dysregulated activation of hypoxia-inducible factor 1-alpha (HIF-1α), all of which are implicated in tissue injury and dysfunction [75].

Moreover, NK-A 17E-233I retained basal respiration compared to Brequinar, suggesting that the depletion of ATP and collapse of membrane potential result from selective inhibition of DHODH rather than broader impairment of the ETC. This mechanistic selectivity may mitigate mitochondrial toxicity in metabolically active tissues, such as the liver.

### Strengths and limitations

This study represents the first successful endeavor since the early 1990 s to identify a novel and effective competitive inhibitor of human DHODH, NK-A 17E-233I, that targets the DHO binding site. Additionally, it elucidates a previously unrecognized effect of Brequinar in stimulating complex II activity in cancer cells. This finding may provide new insights into mitochondrial dysregulation and the metabolic consequences associated with the inhibition of DHODH.

Furthermore, this research provides compelling evidence regarding the significance of the activity of DHODH in POLD2-dependent DNA synthesis. This establishes a mechanistic link between nucleotide metabolism and DNA replication in cancer—an area that has not been previously explored. The expression of POLD2 should be further validated as a biomarker for therapeutic stratification [47, 48]. While this study focuses on comprehensive cellular and mechanistic characterization, the in vivo evaluation of NK-A 17E-233I falls outside its intended scope. Future studies will be necessary to assess its pharmacokinetics and pharmacodynamics in animal models to support its translational development.

## Conclusion

NK-A 17E-233I is a novel, selective competitive inhibitor of human DHODH that specifically targets the natural substrate, DHO, while preserving mitochondrial function and demonstrating efficacy at physiological concentrations of uridine. Its ETC-sparing profile, coupled with a distinct mechanism linked to replication stress, positions it as a promising next-generation chemotype for exploiting metabolic vulnerabilities in cancer. These findings suggest the therapeutic potential of NK-A 17E-233I not only in oncology, but also in autoimmune contexts.

## Supplementary Information

Supplementary Material 1: Supplementary Figure 1, related to Figure 1. A Schematic representation of the prospective virtual screening methodology employed in the current study. *Compounds lacking publications in cancer research. **PAINS and Brenk filters eliminate the following groups: quinones, hydroquinone, thiocarbonyl, thioester, anilines, imines, isolated alkene, quaternary nitrogen, aldehyde, alkyl halide, single bond, triple bond, β-ketoanhydride, peroxide, michael acceptor, polyene, three-membered heterocycle, > 2 esters, coumarine, diketo, nitro group, oxygen-nitrogen, polycyclic aromatic hydrocarbon, phenol ester, keto-keto-β, acyclic C=C-O, hydroxamic acid, phthalimide, and keto-keto-γ. B A summary of the molecular properties of NK-A 17E-233I utilizing SwissADME, STOPLIGHT, and ProtoPRED in silico tools

Supplementary Material 2: Supplementary Figure 2, related to Figure 2. A The viability of cancer cells was assessed in the presence of DMSO or Tx (25 µM) over time using the Trypan Blue exclusion assay. B Representative brightfield images illustrate the effects of Tx (25 µM) on a panel of human cancer and healthy cells. Scale bar = 50 µm. C A dose-response curve depicts the cytotoxicity of BRQ on SW620 colorectal cancer cells after 96 h. Data are presented as mean ± SD (n = 3). D The viability of colorectal cancer cells was evaluated in the presence of DMSO or Tx (25 µM), as well as in the presence of uridine at the specified concentrations, using the CellTiter Glo assay. Data are presented as mean ± SEM (n = 4–6 experiments per condition). Statistical differences were determined using a one-way ANOVA test with Sidak’s multiple comparisons test. E A dose-response curve illustrates the cytotoxicity of Tx on colorectal cancer-derived organoids after 96 h. Data are presented as mean ± SD (n = 3). F Representative images show the effects of Tx at varying concentrations on colorectal cancer patient-derived organoids after 96 h. Scale bar = 100 μm. Tx and BRQ denote NK-A 17E-233I and Brequinar, respectively

Supplementary Material 3: Supplementary Figure 3, related to Figure 3. A Western blot analysis of POLD2 and GAPDH (loading control) from the indicated conditions. B Full western blot membranes from crops in A. C mRNA expression levels, expressed as a fold change, of the specified genes following treatment with DMSO or Tx (25 µM) were analyzed via RT-qPCR (n = 3). Statistical differences were assessed using multiple unpaired t-tests for each gene. D Representative western blots illustrate DHODH, POLD1, and POLD2 protein levels derived from one set of DMSO- or Tx-treated cell extracts (n = 3). α-tubulin and GAPDH served as loading controls. E Full western blot membranes from crops in D. F Graphical representation depicts the downregulation of the orotate metabolite exchange reaction upon treatment with Tx (25 µM). G Analysis of sink reaction flux rates across treated and untreated controls

Supplementary Material 4: Supplementary Figure 4, related to Figure 4. A,B Representative fluorescence confocal images show the effects of DMSO or Tx (25 µM) on HT-29 colorectal cancer cells in terms of mitochondrial activity, assessed by MitoTracker Red (grey) to visualize the mitochondrial membrane potential (A) or ROS production (B) evaluated through CellDeepRed staining (grey). Nuclei were stained with DAPI (blue). Scale bar = 20 µm. Violin plots depict the density of MitoTracker (A) or CellDeepRed (B) per cell (calculated as MitoTracker or ROS intensity divided by the area of each cell). N = 2–4 per condition; n represents MitoTracker or ROS density per cell. In A,B, the data are presented as ‘Superplots’ illustrating the mean of the different replicates (large circles) and the distribution of ‘MitoTracker (in A) or ROS density (in B) per cell analyzed’ (color-coded dots) superimposed as a beeswarm plot. The horizontal line indicates the mean, and a paired two-tailed t-test was employed to ascertain the statistical differences

Supplementary Material 5: Supplementary Figure 5, related to Figure 5. A,B CldU tract lengths at the indicated experimental conditions. Each dot represents one fiber (≥ 140 fibers measured per condition). The horizontal line indicates the median (n = 3). Statistical differences were assessed using the Mann–Whitney test. C Full western blot membranes from crops in Fig. 5. D The percentage of colonies of HCT116 cells treated with DMSO, Tx, VE-821, or the combination of both at the indicated conditions. Bars represent the mean ± SD (n = 5), with dots representing individual dishes. Statistical differences were determined using a one-way ANOVA test with Bonferroni’s multiple comparisons test

Supplementary Material 6: Supplementary Figure 6, related to Figure 6. A Histograms depict the cell cycle distribution of colorectal cancer cells treated with DMSO or Tx (25 µM) for a 48-h period, highlighting the sub-G1 DNA content in red. B The percentage of mitotic cells at various time points from time-lapse assays as shown in Fig. 6. The median is indicated by a horizontal line. Each dot represents the percentage from each time-lapse (n = 6). Statistical differences were evaluated using the Mann–Whitney test. C Representative dot plots from the Annexin V-FITC assay following FACS analysis of colorectal cancer cells in the presence of DMSO or Tx (25 µM), with or without the pan-caspase inhibitor Z-VAD (10 µM) for a 24-h period. D-H Full western blot membranes from crops in Fig. 6

Supplementary Material 7.

Supplementary Material 8.

## Data Availability

The data pertinent to this study are accessible within the article and its supplementary materials. Specifically, the data generated from RNASeq utilized and/or analyzed in this research, including the data employed to reconstruct a computational genome-scale metabolic model, have been deposited at the Zenodo repository (https:/doi.org/10.5281/zenodo.14833856). Additionally, the source code for the genome-scale metabolic modeling is available on GitHub: [https://github.com/Angione-Lab/NK-A-17E-233I-DHODH-Inhibitor-for-the-Treatment-of-Colorectal-Cancer](https:/github.com/Angione-Lab/NK-A-17E-233I-DHODH-Inhibitor-for-the-Treatment-of-Colorectal-Cancer).

## Abbreviations

ADP: Adenosine Diphosphate
AMP: Adenosine Monophosphate
ATP: Adenosine Triphosphate
ATR: Ataxia Telangiectasia and Rad3-related protein
BFCN: Basal Forebrain Cholinergic Neuron
BSA: Bovine Serum Albumin
cDNA: Complementary DNA
CDP: Cytidine Diphosphate
CHK1: Checkpoint Kinase 1
CldU: Chlorodeoxyuridine
CMP: Cytidine Monophosphate
CTP: Cytidine Triphosphate
DAPI: 4′,6-Diamidino-2-Phenylindole
DHO: Dihydroorotate
DHODH: Dihydroorotate Dehydrogenase
DMEM: Dulbecco’s Modified Eagle Medium
DMSO: Dimethyl Sulfoxide
DR5: Death Receptor 5 (TRAIL Receptor 2)
EDTA: Ethylenediaminetetraacetic Acid
ETC: Electron Transport Chain
FACS: Fluorescence-Activated Cell Sorting
FBS: Fetal Bovine Serum
FCCP: Carbonyl Cyanide-p-trifluoromethoxyphenylhydrazone
FMN: Flavin Mononucleotide
FMNH_2_: Dihydro FMN
FSC: Forward Scatter
FVA: Flux Variability Analysis
GDP: Guanosine Diphosphate
GMP: Guanosine Monophosphate
GPRs: Gene-Protein-Reaction Rules
GSH: Reduced Glutathione
GSMM: Genome-Scale Metabolic Model
GSSG: Oxidized Glutathione
GTP: Guanosine Triphosphate
HDFa: Human Dermal Fibroblasts
HEKa: Human Epidermal Keratinocytes
HEPES: 4-(2-Hydroxyethyl)-1-Piperazineethanesulfonic Acid
HFF: Human Foreskin Fibroblasts
HKGS: Human Keratinocyte Growth Supplement
HPLC–MS/MS: High-Performance Liquid Chromatography coupled with Tandem Mass Spectrometry
HU: Hydroxyurea
IC_50_: Half-maximal Inhibitory Concentration
IdU: Iododeoxyuridine
KEGG: Kyoto Encyclopedia of Genes and Genomes
*K*_*i*_: Inhibition Constant
*K*_*m*_: Michaelis-Menten Constant
LMA: Low Melting Agarose
MAP2: Microtubule-Associated Protein 2
MOMA: Minimization Of Metabolic Adjustment
nanoDSF: Nano-Differential Scanning Fluorimetry
OCR: Oxygen Consumption Rate
OXPHOS: Oxidative Phosphorylation
PARP: Poly(ADP-ribose) Polymerase
PBS: Phosphate-Buffered Saline
PFA: Paraformaldehyde
PI: Propidium Iodide
PMT: Photomultiplier Tube
POLD2: DNA Polymerase Delta Subunit 2
PVDF: Polyvinylidene Fluoride
qPCR: Quantitative Polymerase Chain Reaction
QSIR: Quantitative Structure Interference Relationship
RLU: Relative Light Unit
RMSD: Root Mean Square Deviation
rMTA: Robust Metabolic Transformation Analysis
RNASeq: RNA Sequencing
ROS: Reactive Oxygen Species
RT-qPCR: Reverse Transcription Quantitative PCR
SD: Standard Deviation
SDS: Sodium Dodecyl Sulfate
SDS-PAGE: SDS-Polyacrylamide Gel Electrophoresis
SEM: Standard Error of the Mean
siRNA: Small Interfering RNA
SSC: Side Scatter
STR: Short Tandem Repeat
TBS-T: Tris-Buffered Saline with Tween-20
TCA: Trichloroacetic Acid
TLS: Translesion Synthesis
*T*_*m*_: Melting Temperature
TPSA: Topological Polar Surface Area
TSA: Thermal Stability Assay
UDP: Uridine Diphosphate
UMP: Uridine Monophosphate
UTP: Uridine Triphosphate
ΔG: Gibbs Free Energy of Binding
ΔΨm: Mitochondrial Membrane Potential
